# *Rhus coriaria* (Sumac) induces autophagic cell death and inhibits mTOR, p38MAPK and STAT3 pathways in 5fluorouracil-resistant colorectal cancer cells

**DOI:** 10.3389/fphar.2025.1542204

**Published:** 2025-03-19

**Authors:** Zohra Nausheen Nizami, Mazoun Al Azzani, Samah Khaldi, Adil Farooq Wali, Rym Magramane, Shamaa Abdul Samad, Ali H. Eid, Kholoud Arafat, Yusra Al Dhaheri, Samir Attoub, Rabah Iratni

**Affiliations:** ^1^ Department of Biology, College of Science, United Arab Emirates University, Al-Ain, United Arab Emirates; ^2^ Department of Pharmaceutical Chemistry, RAK College of Pharmacy, RAK Medical and Health Sciences University, Ras Al Khaimah, United Arab Emirates; ^3^ Department of Basic Medical Sciences, College of Medicine, QU Health, Qatar University, Doha, Qatar; ^4^ Department of Pharmacology and Therapeutics, College of Medicine and Health Sciences, United Arab Emirates University, Al-Ain, United Arab Emirates

**Keywords:** 5-fluorouracil, chemoresistance, colorectal cancer, apoptosis, autophagy, Survivin

## Abstract

**Introduction:**

Colorectal cancer is a leading cause of cancer related-death worldwide, and resistance to 5-fluorouracil (5FU, a key component of chemotherapy regimens, is a major clinical concern. We have previously elucidated the effects of *Rhus coriaria* ethanolic extract (RCE) in triple-negative breast cancer, CRC, and pancreatic cancer cells. Here, we explored the anticancer effects of RCE in parental (HCT-116-WT) and 5FU-resistant HCT-116 (HCT-116-5FU-R) CRC cells.

**Methods:**

MTT assay was used to assess cell viability. Muse analyzer was used to assess cell viability, cell cycle distribution, and apoptosis. Additionally, colony formation and growth assays and western blots were performed. *In vivo* effects of RCE were assessed by an *in ovo* chick embryo tumor growth assay.

**Results:**

We found that RCE inhibited the viability and colony formation and growth capacities of HCT-116-WT and HCT-116-5FU-R cells. The antiproliferative effects were attributed to DNA damage-mediated impairment of cell cycle at S phase, and induction of Beclin-1-independent autophagy in both cell lines. Mechanistically, inhibition of the mTOR, STAT3 and p38 MAPK pathways was implicated in the latter. Additionally, RCE induced caspase-7-independent apoptosis in HCT-116-WT cells. However, HCT-116-5FU-R cells were resistant to apoptosis through upregulation of survivin, and downregulation of Bax. Using autophagy and proteasome inhibitors, we clarified that autophagy and the proteasome pathway contributed to RCE-mediated cell death in HCT-116-WT and HCT-116-5FU-R cells. Lastly, we confirmed RCE inhibited the growth of both HCT-116-WT and HCT-116-5FU-R xenografts in a chick embryo model.

**Discussion:**

Collectively, our findings highlight that RCE is a source of phytochemicals that can be used as anticancer agents for 5FU-resistant CRC.

## 1 Introduction

In 2020, colorectal cancer (CRC) was the third most diagnosed cancer and the second leading cause of cancer related-deaths worldwide (Sung et al., 2021). Moreover, the number of CRC cases are expected to increase by 65% in 2040 (Xi and Xu, 2021). While surgical approaches are the gold standard for stage I CRC, adjuvant chemotherapy (FOLFOX, CapeOX, FOLFIRI, and FOLFOXIRI) is used starting from stage II CRC (Dekker et al., 2019; PDQ^®^ Adult Treatment Editorial Board, 2023). 5-Fluorouracil (5FU), a pyrimidine analogue, is an important component of all the above mentioned chemotherapy regimens used clinically for CRC treatment. 5FU exerts its cytotoxic effects mainly through its active metabolite fluorodeoxyuridine monophosphate, which inhibits the activity of thymidylate synthase (TS), an enzyme involved in DNA biosynthesis (Ghafouri-Fard et al., 2021). This results in impairment of DNA synthesis and repair, and ultimately DNA damage-induced cell death. A secondary mechanism of action of 5FU is misincorporation of other active metabolites into RNA and DNA, which impairs the processing of precursor RNA, disrupts post-transcriptional modification of tRNA, and inhibits splicing of mRNA (Vodenkova et al., 2020).

5FU resistance through intrinsic or acquired mutations undermines the effectiveness of 5FU-based chemotherapeutic regimens and is a major clinical concern for CRC treatment. Mechanisms of 5FU resistance include TS polymorphisms that increase its expression and/or activity, overexpression of dihydropyrimidine dehydrogenase, which catabolizes 5FU to its inactive metabolite, underexpression of thymidine phosphorylase, which catalyzes the conversion of 5FU to an intermediate required for the production of its active metabolite, among others (Azwar et al., 2021; Sethy and Kundu, 2021). Other mechanisms include resistance to DNA damage as a result of aberrations in DNA repair proteins (increased expression and/or activity), and overexpression of transporter pumps, such as ATP-binding cassette transporters (Azwar et al., 2021; Sethy and Kundu, 2021; Blondy et al., 2020). In fact, autophagy, apoptosis, epithelial–mesenchymal transition, and non-coding RNAs have also been implicated in 5FU resistance (Azwar et al., 2021; Sethy and Kundu, 2021; Blondy et al., 2020).

In recent years, plant-based therapeutic approaches have gained popularity in cancer research. Specifically, phytochemicals have emerged as promising anti-cancer drugs due to their intrinsic anti-cancer activity (Choudhari et al., 2020; Redondo-Blanco et al., 2017). Increasing number of phytochemicals are being investigated for their anti-cancer activity for synergistic or additive effects with conventional chemotherapeutic drugs. For example, curcumin, the main active ingredient of *Curcuma longa* (Turmeric) extract, and its derivatives are presently in Phase I and II clinical trials for CRC (NCT01859858, NCT01294072, NCT01333917, NCT02439385) (Nizami et al., 2023). Curcumin has also been shown to synergize with and increase the efficacy of 5FU-oxaliplatin therapy in resistant colon cancer cell lines and explants (Redondo-Blanco et al., 2017).

Our lab previously reported the anti-cancer effects of *Rhus coriaria* fruit ethanolic extract (RCE). *Rhus coriaria* L. or Sumac, is a plant native to the Mediterranean region, whose dried fruits are used as spice (Elagbar et al., 2020; Rayne and Mazza, 2007). *R. coriaria* fruits are also used traditionally for medicinal purposes, including for treatment of ulcers, diarrhea, urinary tract infections, sore throat, and indigestion (Elagbar et al., 2020). Our lab was the first to investigate the anti-cancer activity of RCE and reported that RCE inhibited the proliferation of triple-negative breast cancer (TNBC) cells (MDA-MB-231) by inducing G1 cell cycle arrest and senescence. We also reported that RCE induced DNA damage that mediated Beclin-1-dependent autophagy through activation of the p38 and ERK1/2 pathways (El Hasasna et al., 2015). Later, we reported that RCE decreased the migration and invasion capacity of TNBC cells by downregulating the expression of matrix metalloproteinase nine and prostaglandin E2, and cancer metastasis-associated inflammatory cytokines tumor necrosis factor-α and interleukins −6 and −8, which was confirmed in an *in vivo* chick embryo model. RCE was also found to inhibit angiogenesis by downregulating the expression of vascular endothelial growth factor. These effects were attributed to RCE-mediated inhibition of the NFκB, STAT3, and nitric oxide signaling pathways (El Hasasna et al., 2016).

To the best of our knowledge, we are the only group to report on the effects of RCE against CRC. We reported that RCE inhibited the proliferation of CRC cells (HT-29 and Caco-2) as well as tumor growth in mouse xenografts. Additionally, RCE was found to induce Beclin-1-independent autophagy through targeted proteasome-mediated degradation of Beclin-1 and mammalian target of rapamycin (mTOR) and AKT, negative regulators of autophagy. Further, autophagy was noted to be an early event that triggered Caspase-7-dependent apoptosis, as RCE targeted pro-caspase-3 to proteasome-mediated degradation, which was concomitant with increased global ubiquitination (Athamneh et al., 2017). Although the anti-cancer activity of RCE against CRC cells has been characterized, its effects against 5FU-resistant CRC have not been reported. Hence, the present study aimed to characterize the anti-cancer activity of RCE against 5FU-resistant CRC using parental/5FU-sensitive (HCT-116-WT) and 5FU-resistant (HCT-116-5FU-R) CRC cells.

## 2 Materials and Methods

### 2.1 Reagents

Primary antibodies against phosphorylated mTOR (p-mTOR, Ser2448; 2971), mTOR (2972), p-STAT3 (Tyr705; 9131), STAT3 (9139), Caspase 8 (9746), Caspase 7 (9492), Caspase 3 (9662S), Beclin-1 (3495), CDK2 (2546), LC3A/B I/II (12741), p27 (3686S), p-p38MAPK (Thr180/Tyr182; 4511S), p38 MAPK (8690S), and Bax (2772S) were obtained from Cell Signaling Technology (Danvers, MA, United States); against Caspase 9 (05-572), Cyclin D1 (04-1151), Cyclin B1 (05-373), Rb (05-377), p-Rb (ABC132), and phosphorylated Histone H2A.X (Ser139, γH2AX; 05–636) were obtained from Millipore (Burlington, MA, United States); against p62 (Ab101266) and cleaved PARP (Ab4830) were obtained from Abcam (Cambridge, United Kingdom); against Survivin (sc-17779) were obtained from Santa Cruz Biotechnology (Dallas, TX, United States); against full length and cleaved PARP (556494) and p21 (556431) from BD Biosciences (Franklin Lakes, NJ, United States). Horseradish peroxidase (HRP)-conjugated goat anti-mouse (sc-2005) and goat anti-rabbit (sc-2030) antibodies, and HRP-conjugated antibodies against β-actin (sc-47778 HRP) were obtained from Santa Cruz Biotechnology. Bortezomib and MG-132 were obtained from Cell Signaling Technology, 3-methyl adenine (3-MA) and Z-VAD-FMK from Millipore, and 5FU and chloroquine (CQ) from Sigma-Aldrich (Saint-Quentin Fallavier, France).

### 2.2 Preparation of RCE extract and treatment

RCE was prepared as described previously (Al Dhaheri et al., 2013). Briefly, fruits of *R. coriaria* L. were collected from a private farm in Ma’rakeh, Tyre, Lebanon (33°16′35.59″N and 35°19′02.89″E) with the approval of the owner. The dried fruit (10 g) was ground and the powder was resuspended in 70% absolute ethanol and incubated in the dark for 72 h at 4°C with constant agitation. Subsequently, the solution was filtered using a glass sintered funnel, the filtrate was evaporated using a rota-vapor, and the resultant residue was stored in vacuum for 2–3 h, following which its mass was recorded. The red residue was stored at −20°C and was reconstituted in 70% absolute ethanol as required for use in experiments.

### 2.3 HPLC-MS analysis of RCE

The phytochemical constituents of RCE were analyzed using HPLC-MS approach with the Agilent Technologies 6420 Triple Quadrupole platform [(Agilent Technologies, Santa Clara, CA, United States). To this end, RCE was filtered through a 0.45 μm syringe filter prior to analysis. The LC-MS system used was comprised of the following components (all from Agilent Technologies]: an Agilent EclipsePlus-C18 column (1.8 μm particle size, 2.1 mm × 50 mm length) maintained at 35°C, a tunable UV-Vis detector, and the 6420 Triple Quadrupole LC/MS System. The mobile phases included 0.1% formic acid (A) and acetonitrile (B), with the following gradient profile at a flow rate of 0.2 mL/min: 0–2.5 min, 0% B; 2.5–15 min, 20%–100% B; 15–18 min, 100% B; and 18–25 min, 5% B. Electrospray ionization was employed in the positive polarity mode. The capillary voltage was set to +4 kV and the nebulizer pressure to 45 psi. Drying gas was passed through at a rate of 11 L/min, maintaining a drying temperature of 325 °C. A mass detection range of 100–000 Da was achieved, which facilitated the characterization of RCE constituents.

HPLC-MS analysis (Supplementary Figure S1A) revealed several peaks, which corresponded to the presence of the following eight phytochemicals in RCE (Table 1; Supplementary Figure S1B): Quinic acid (MW: 192.17 g/mol; RT: 1.76 min), Gallic Acid (MW: 170.12 g/mol; RT: 3.12 min), 1-O-(4-Coumaroyl)-beta-D-glucose (MW: 326.3 g/mol; RT: 11.98 min), Quercetin (MW: 302.23 g/mol; RT: 16.15 min), Digallic Acid (MW: 332.01 g/mol; RT: 16.66 min), Sespendole (MW: 519.7 g/mol; RT: 17.11 min), Genistin (MW: 432.4 g/mol; RT: 19.59 min), and Phloretin 2′-glucoside (MW: 436.42 g/mol; RT: 23.01 min).

**TABLE 1:** Phytochemical constituents of RCE identified through HPLC-MS analysis.

S. No	RT (min)	Phytochemical	Molecular formula	Molecular Weight (g/mol)	Chemical structure
1	1.76	Quinic acid	C_7_H_12_O_6_	192.17	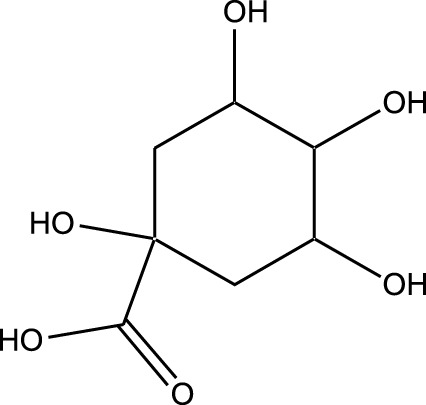
2	3.12	Gallic Acid	C_7_H_6_O_5_	170.12	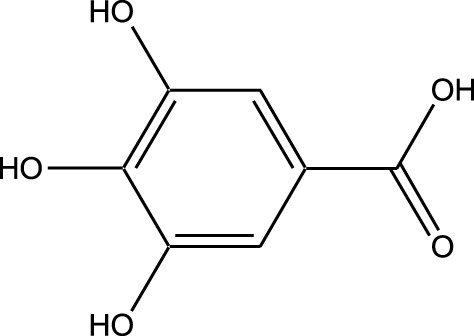
3	11.98	1-O-(4-Coumaroyl)-beta-D-glucose	C_15_H_18_O_8_	326.30	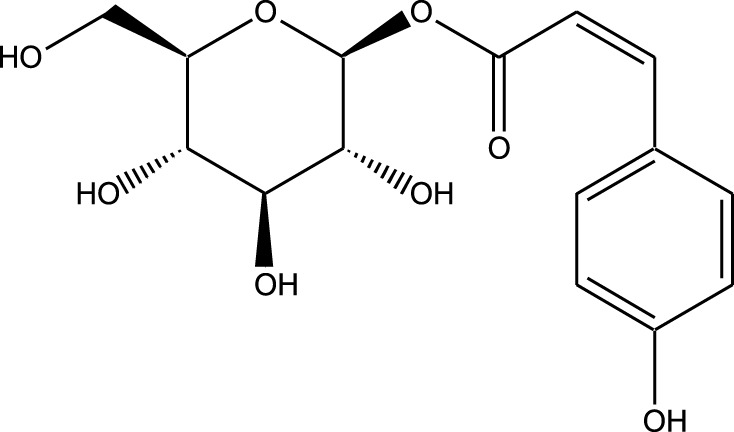
4	16.15	Quercetin	C_15_H_10_O_7_	302.23	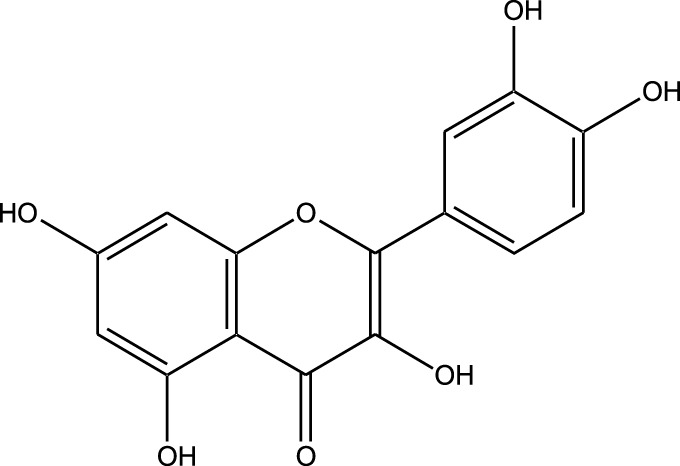
5	16.66	Digallic Acid	C_14_H_10_O_9_	332.01	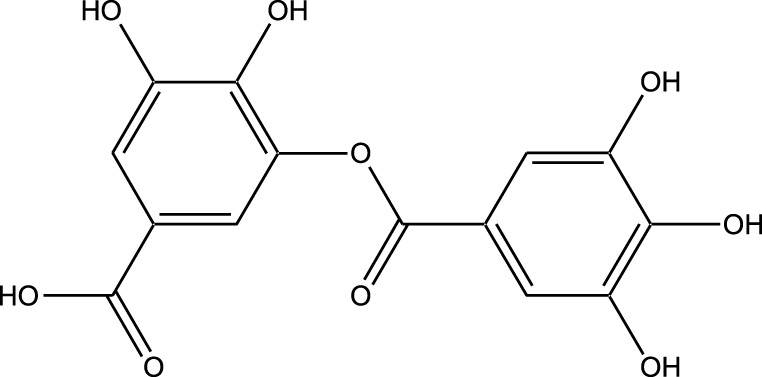
6	17.11	Sespendole	C_33_H_45_NO_4_	519.7	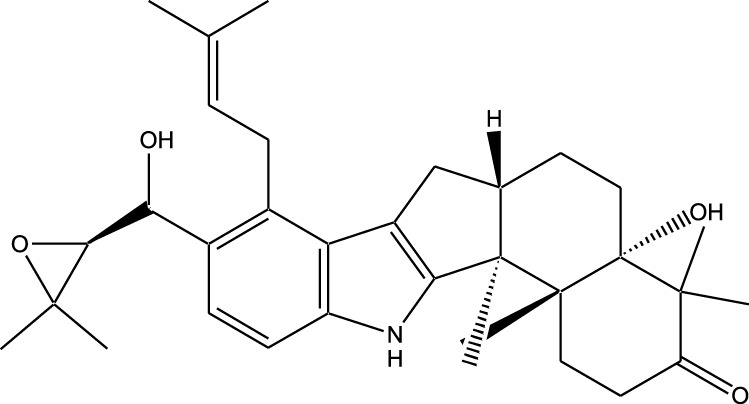
7	19.59	Genistin	C_21_H_20_O_10_	432.40	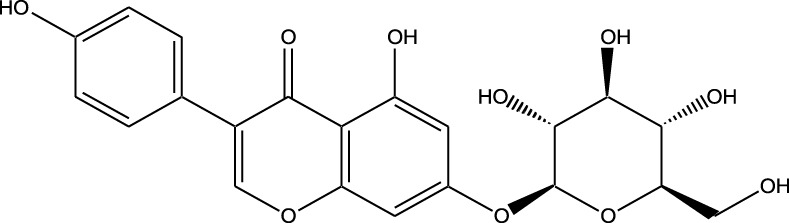
8	23.01	Phloretin 2′-glucoside	C_21_H_24_O_10_	436.42	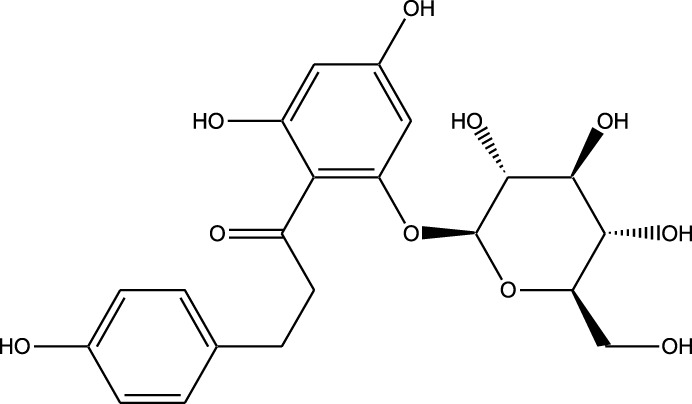

RT, retention time.

### 2.4 Cell culture and treatment

Human CRC cell lines, parental HCT-116-WT and HCT-116-5FU-R (a kind gift from American University of Beirut, Beirut, Lebanon) were cultured in Dulbecco’s Modified Eagle Medium (DMEM; Hyclone, Cramlington, United Kingdom) supplemented with 10% heat-inactivated fetal bovine serum (Thermo Fisher Scientific, Waltham, MA, United States) and 100 U/mL penicillin/streptomycin/gentamicin (Hyclone). Cell lines were maintained in a humidified 5% CO2 environment at 37°C (Binder, Tuttlingen, Germany).

HCT-116-5FU-R cells were derived from parental HCT-116-WT cells by long-term culture (8 months) in increasing concentrations of 5FU (0.1–40 µM); however, the exact mechanism of 5FU resistance in these cells has not been clarified (Abdel-Samad et al., 2018; Hamze et al., 2023).

CRC cells were treated with or without RCE at the indicated concentrations for 24 and/or 48 h. For experiments with autophagy, apoptosis, and/or proteasome inhibitors, CRC cells were pre-treated for 2 h and then treated with media containing RCE at the indicated concentration with the inhibitor.

### 2.5 Measurement of cell viability

#### 2.5.1 MTT assay

Cell viability was measured using the MTT assay kit (Abcam, Cambridge United Kingdom), which measures metabolic activity as an index of formazan formation, as per the manufacturer’s instructions. Briefly, CRC cells were seeded in triplicate in 96-well plates (Corning, Corning, NY, United States) at a density of 5,500 cells/well and 7,000 cells/well for HCT-116-WT and HCT-116-5FU-R, respectively, and cultured overnight. CRC cells were subsequently treated with the indicated concentrations of RCE for 24 and 48 h, and with the indicated concentrations of 5FU for 24, 48, and 72 h, respectively. Absorbance was measured using a microplate reader (Platos R 496, AMEDA Labordiagnostik GmbH, Graz, Austria). Data are presented as percentage of the cell viability (%) of treated cells with respect to the control cells.

#### 2.5.2 Muse cell count assay

MTT assay measures cell metabolic activity and cannot differentiate between reduction in metabolic activity due to cytostatic or cytotoxic effects. Hence, we assessed cell viability using the Muse^®^ Count and Viability Kit (Luminex Corp., Austin, TX, United States), which differentially stains viable and non-viable cells based on their permeability to DNA-binding dyes, as per the manufacturer’s instructions. Briefly, CRC cells were seeded in triplicate in 12-well plates (Corning) at a density of 5.5 × 10^4^ cells/well and 7.5 × 10^4^ cells/well for HCT-116-WT and HCT-116-5FU-R, respectively, and cultured overnight prior to treatment with the indicated concentrations of RCE for 24 h. The cell pellet was collected following trypsinization, resuspended in complete DMEM media, and diluted as per the kit’s instructions. Viable cells were then counted using the Muse™ Cell Analyzer (Luminex Corp., Austin, TX, United States). Cells were collected and counted on the day of treatment to estimate the approximate number of cells, which was considered as Day 0. Data are presented both as the number of viable cells on Days 0 and 1 following treatment. Of note, Muse cell count assay was performed to clarify the potent cytotoxic effects observed by cell viability assay (MTT) at 24 h post-treatment with RCE.

### 2.6 Colony formation and growth assays

#### 2.6.1 Colony formation assay

HCT-116-WT and HCT-116-5FU-R cells were seeded at a density of 450 and 550 cells/well, respectively, in 6-well plates (Corning). Cells were treated with the indicated concentrations of RCE on day 3 (2–4 cells stage of colonies) for another 8 days. The formed colonies were fixed on day 11 using a 10% formaldehyde solution (4% para-formaldehyde, pH 7.0) (Sigma-Aldrich) for 15 min and stained with a 0.01% (w/v) crystal violet solution for 30 min. Fresh media or treatment media was replaced every 3 days. The stained colonies were photographed and the number of colonies was calculated using ImageJ software (National Institutes of Health, Bethesda, MD, United States).

#### 2.6.2 Colony growth assay

HCT-116-WT and HCT-116-5FU-R cells were seeded at a density of 450 and 550 cells/well, respectively, in duplicate 6-well plates (Corning). Both plates were allowed to grow for 11 days until small-to-medium sized colonies were observed. The formed colonies on plate 1 were fixed and stained, as described above, to serve as a base line for the number of colonies on the day of treatment. Colonies on plate 2 were treated with the indicated concentrations of RCE for another 4 days, and then fixed and stained. Fresh media or treatment media was replaced every 3 days. The stained colonies were photographed and the number of colonies was calculated using ImageJ software (National Institutes of Health, Bethesda, MD, United States).

### 2.7 Annexin V apoptosis assay

RCE-mediated induction of apoptosis was assessed using the Muse^®^ Annexin V Dead Cell kit (Millipore, Billerca, MA, United States) as per the manufacturer’s instructions. Briefly, CRC cells were seeded in triplicate in 12-well plates (Corning) at a density of 5.5 × 10^4^ cells/well and 7.5 × 10^4^ cells/well for HCT-116-WT and HCT-116-5FU-R, respectively. Cells were cultured overnight before treatment with the indicated concentrations of RCE for 48 h. Both detached and adherent cells were collected and incubated with Annexin-V and 7-AAD for 20 min in the dark at room temperature. The number of live and apoptotic (early and late) cells were counted using the Muse™ Cell Analyzer (Luminex Corp.).

### 2.8 Cell cycle analysis

Cell cycle distribution following RCE treatment was assessed using the Muse^®^ Cell Cycle Kit (Luminex Corp.) as per the manufacturer’s instructions. Briefly, CRC cells were seeded in triplicate in 60 mm dishes (Corning) at a density of 2 × 10^5^ cells/dish and 2.5 × 10^5^ cells/dish for HCT-116-WT and HCT-116-5FU-R, respectively. Cells were cultured overnight before treatment with the indicated concentrations of RCE for 48 h, following which the cells were trypsinized and pelleted by centrifugation. The cell pellet was washed and resuspended in cold 1× phosphate-buffered saline (PBS) and then fixed with an equal volume of 100% absolute ethanol by incubation at −20°C for a minimum of 3 h. On the day of flow cytometric analysis, the cells were pelleted, washed with cold 1× PBS, and stained for 30 min in the dark with the Muse^®^ Cell Cycle Kit (Luminex Corp.). Cells were counted and analyzed with the Muse™ Cell Analyzer (Luminex Corp.), and cell cycle distribution was determined using the FlowJo 10.0 Software (BD Biosciences).

### 2.9 Western blot

CRC cells were seeded in duplicate or triplicate in 100 mm dishes (Corning) at a density of 2 × 10^6^ cells/dish and 2.2 × 10^6^ cells/dish for HCT-116-WT and HCT-116-5FU-R, respectively. Cells were cultured overnight before treatment with the indicated concentrations of RCE for 48 h. Following treatment, the non-adherent cells in the culture media were pelleted by centrifugation and the adherent cells were trypsinized and pelleted. The total cell pellet was then lysed in 100 µL RIPA Buffer (Thermo Fisher Scientific) supplemented with protease and phosphatase inhibitor cocktails (Roche, Basel, Switzerland). The cell lysate was sonicated and centrifuged, and the supernatant was collected as the protein lysate and quantified using the Pierce™ BCA Protein Assay Kit (Thermo Fisher Scientific, Waltham, MA, United States). Proteins (30 µg) were separated on 6%–15% sodium dodecyl sulfate-polyacrylamide gel electrophoresis gels and transferred onto methanol-activated PVDF membranes (Millipore). The membranes were blocked with 5% low fat milk in Tris-buffered saline with 0.1% Tween 20 (TBS-T) (blocking buffer) for 1 h at room temperature, incubated with the indicated primary antibodies in blocking buffer overnight at 4°C, and with the corresponding HRP-conjugated secondary antibodies in 3% low fat milk in TBS-T at room temperature for 1 h. Probed membranes were visualized on the C-DiGit imaging system (Licor, Lincoln, NE, United States) using the SuperSignal™ West Pico PLUS Chemiluminescent Substrate (Thermo Fisher Scientific).

### 2.10 *In ovo* chick embryo tumor growth assay

The *in ovo* chick embryo tumor growth assay was performed as described previously (Alsamri et al., 2019). Briefly, fertilized chicken eggs were incubated at 37.5°C and 50% humidity. On embryonic day 3 (E3), the chorioallantoic membrane (CAM) was dropped by aspirating 1.5–2 mL of albumin, through a hole opposite to the round wide egg side, and a 1 cm^2^ window was cut in the eggshell above the CAM and sealed with a semipermeable adhesive film (Suprasorb^®^ F). On E9, CRC cells were detached by trypsinization, washed with complete medium, and resuspended in 50% Matrigel. A 100-µL inoculum (50 µL Matrigel and 50 µL 0.9% saline) of 5 × 10^6^ HCT-116-WT or HCT-116-5FU-R cells were inoculated onto the CAM of each egg. The eggs were randomized in two groups (control and treated) with 16 eggs per group. The tumors were detectable on E11, and were treated with 100 µL of the vehicle (0.9% saline with 0.01% of ethanol) or RCE (50 mg/kg prepared in 0.9% saline). The treatment was repeated on E13 and E15. On E17, the upper portion of the CAM was removed, washed with PBS, and then the tumors were carefully cut away from normal CAM tissues and weighed to determine the impact of RCE on tumor growth. The *in ovo* tumor xenograft experiments were performed in accordance with the protocol approved by the United Arab Emirates University Animal Ethics Committee (Permit No. ERA_2022_2154 on 14/03/2023).

### 2.11 Statistical analysis

Data was analyzed using the GraphPad Prism 10 (La Jolla, CA, United States) and presented as the mean ± standard error of mean (SEM). Differences between groups were analyzed using one-way ANOVA followed by Fisher’s LSD test. The unpaired t-test was applied to evaluate the difference between the two groups. A p-value <0.05 was considered statistically significant. All experiments were repeated a minimum of three times, except for the CAM tumor growth assay or stated otherwise.

## 3 Results

### 3.1 *Rhus coriaria* inhibits the viability of parental and 5FU-resistant HCT-116 CRC cells

To examine the anti-cancer activity of RCE against parental and 5FU-resistant CRC, we first confirmed the sensitivity of HCT-116-WT and HCT-116-F-FU-R cells to 5FU. To this end, both cell lines were treated with increasing concentrations of 5FU (0, 5, 10, 20, 40 µM) for 24, 48, and 72 h. Consistently, we verified that HCT-116-WT cells were sensitive to 5FU as evidenced by the concentration- and time-dependent decrease in cell viability (Supplementary Figure S2A). As expected, HCT-116-5FU-R cells were resistant to 5FU as evidenced by the lack of effect on cell viability even at 40 µM 5FU following 72 h of treatment (Supplementary Figure S2B).

**FIGURE 1:**
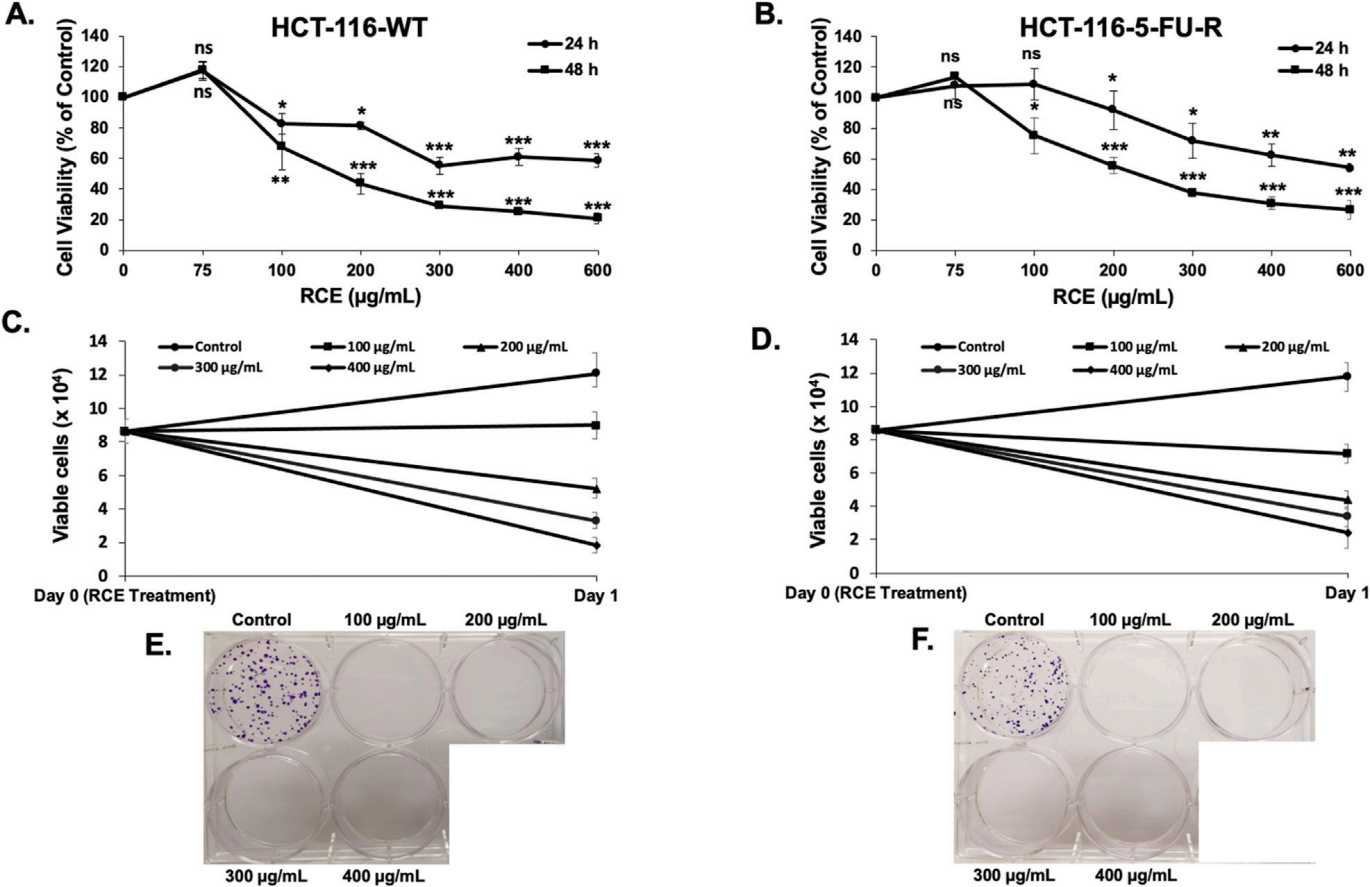
*Rhus coriaria* inhibits the viability and colony formation of parental and 5FU-resistant HCT-116 CRC cells. **(A)** HCT-116-WT and **(B)** HCT-116-5FU-R CRC cells were treated with varying concentrations of RCE (0, 100, 200, 300, 400, and 600 μg/mL) for 24 and 48 h, following which cell viability was assessed using MTT assay as described in the Materials and Methods section. Data is presented as the mean ± SEM; n = 3–4, in triplicate. ns: not significant, *p < 0.05, **p < 0.005, ***p < 0.001 (vs. 0 μg/mL); one way ANOVA followed by Fisher’s LSD test. **(C)** HCT-116-WT and **(D)** HCT-116-5FU-R CRC cells were treated with varying concentrations of RCE (0, 100, 200, 300, and 400 μg/mL) for 24 h, following which cell viability was assessed using MUSE Cell Analyzer (Luminex Corp., Austin, TX, United States) as described in the Materials and Methods section. Data is presented as the mean ± SEM; n = 4, in duplicate. **(E)** HCT-116-WT and **(F)** HCT-116-5FU-R CRC cells were seeded in duplicate 6-well plates at low densities and treated on colony day 3. The colonies were treated for 8 days and stained at colony day 11 (post treatment day 8).

Next, we examined the anti-cancer effect of RCE (0, 100, 200, 300, 400, and 600 μg/mL) against parental and 5FU-resistant HCT-116 CRC cells using MTT assay, which evaluates cellular metabolic activity, more specifically, mitochondrial activity. We found that RCE inhibited the viability of HCT-116-WT cells (Figure 1A) and HCT-116-5FU-R cells (Figure 1B) in a concentration- and time-dependent manner. The IC_50_ values for HCT-116-WT cells were 246.5 and 180.5 μg/mL at 24 and 48 h, respectively. Whereas, the IC_50_ values for HCT-116-5FU-R cells were 247.1 and 216.7 μg/mL at 24 and 48 h, respectively. Importantly, based on the percentage cell viability following 48 h of RCE treatment, the anti-proliferative effect of RCE was comparable between the two cell lines, especially at higher concentrations (400 and 600 μg/mL).

To further validate the results of MTT assay, cell viability was also assessed using a flow cytometry-based assay that differentially stains viable and dead cells based on their permeability to DNA-binding dyes, as described in the Materials and Methods section. We found that consistent with the results of the MTT assay, RCE decreased the number of viable HCT-116-WT (Figure 1C) and HCT-116-5FU-R (Figure 1D) cells in a concentration-dependent manner following 24 h of RCE treatment, when compared to the number of cells counted on the day of treatment (denoted as day 0). Collectively, these findings suggest that RCE induces cell death and/or inhibits the proliferation of both parental and 5FU-resistant HCT-116 CRC cells.

### 3.2 *Rhus coriaria* inhibits the colony formation and growth of parental and 5FU-resistant HCT-116 CRC cells

**FIGURE 2:**
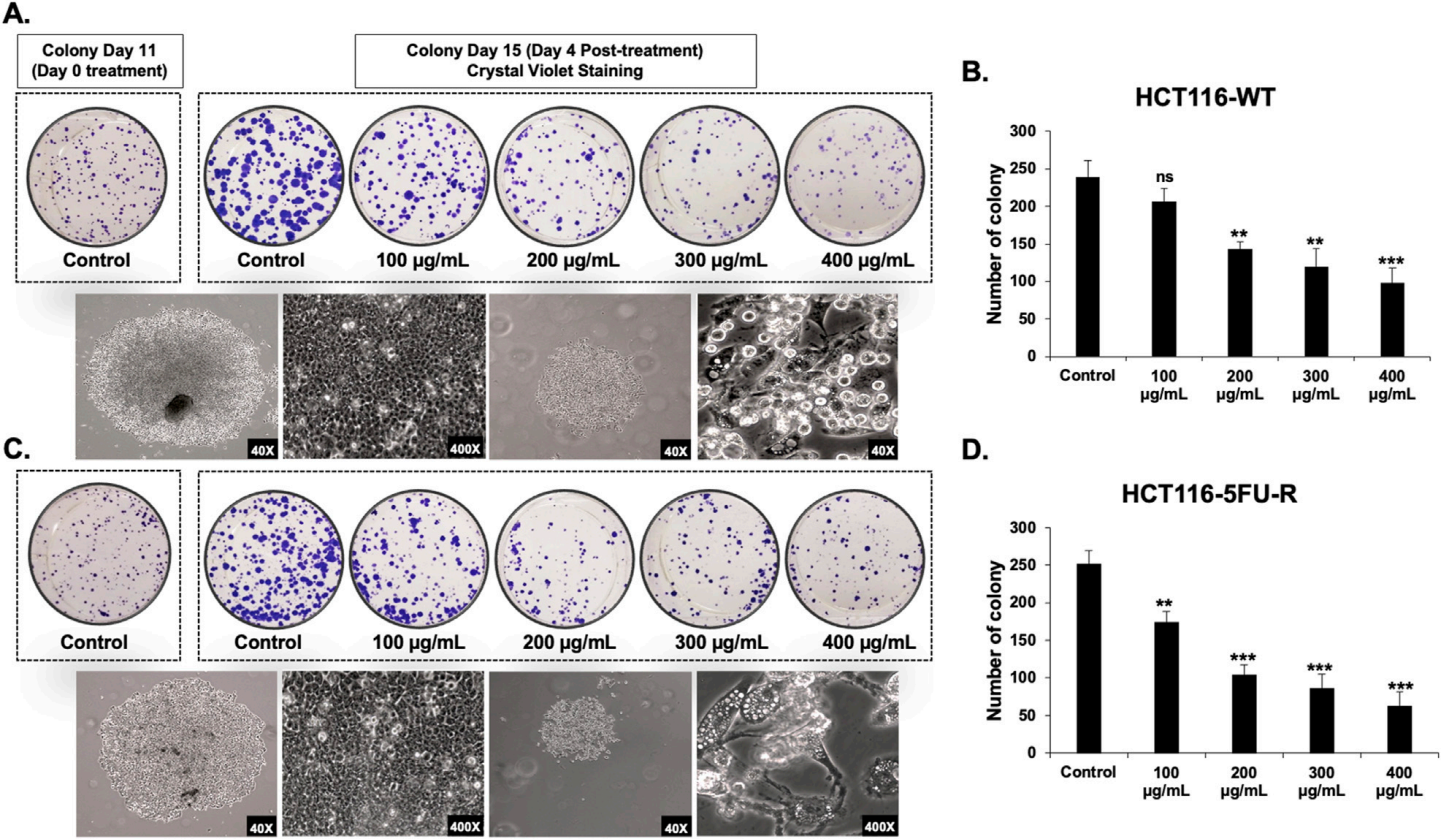
*Rhus coriaria* inhibits the growth of parental and 5FU-resistant HCT-116 CRC colonies. HCT-116-WT and HCT-116-5FU-R CRC cells were seeded in duplicate 6-well plates at low densities, and cultured for 11 days to allow colony formation. The formed colonies were treated on colony day 11 and stained at colony day 15 (post treatment day 4). Representative images of **(A)** HCT-116-WT and **(C)** HCT-116-5FU-R colonies following staining with crystal violet, and of control and RCE (200 μg/mL)-treated colonies under light microscopy. Quantitative analysis of the number of colonies of **(B)** HCT-116-WT and **(D)** HCT-116-5FU-R CRC at post-treatment day 4 (colony day 15) Data is presented as the mean ± SEM; n = 3–4. ns: not significant, *p < 0.05, **p < 0.005, ***p < 0.001 (vs. Control); one-way ANOVA followed by Fisher’s LSD test.

Next, we assessed the anti-proliferative effect of RCE on parental and 5FU-resistant CRC cells by assessing its effect on the formation of CRC colonies. To this end, HCT-116-WT and HCT-116-5FU-R cells were seeded at low densities and treated with varying concentrations of RCE (0, 100, 200, 300, and 400 μg/mL) when the colonies were at the 2–4 cell stage. RCE completely abrogated the colony formation ability of both HCT-116-WT (Figure 1E) and HCT-116-5FU-R (Figure 1F) cells, even at 100 μg/mL. Subsequently, we assessed the effect of RCE on the growth of formed parental and 5FU-resistant CRC colonies. To this end, HCT-116-WT and HCT-116-5FU-R cells were seeded at low densities and cultured for 11 days to facilitate colony formation. Formed colonies were then treated with varying concentrations of RCE (0, 100, 200, 300, and 400 μg/mL) for 4 days. We found that compared to the colonies on the day of treatment (colony day 11; day 0 treatment), RCE inhibited the subsequent growth of both HCT-116-WT (Figure 2A) and HCT-116-5FU-R (Figure 2C) colonies in a concentration-dependent manner (colony day 15; day 5 post-treatment); whereas, the colonies in the untreated controls of both cell lines continued to grow. This is further confirmed by the significant concentration-dependent decrease in the number of HCT-116-WT (Figure 2B) and HCT-116-5FU-R (Figure 2D) colonies. Additionally, microscopic examination of the treated colonies showed that RCE treatment (200 μg/mL) affected the integrity of the colonies as more diffused/fragmented colonies with massive cytoplasmic vacuolation and cell death were noted in both cell lines (Figures 2A, C). Collectively, these findings provide further evidence to the antiproliferative effect of RCE against parental and 5FU-resistant HCT-116 CRC cells.

### 3.3 *Rhus coriaria* induces DNA damage-mediated S phase cell cycle arrest in parental and 5FU-resistant HCT-116 CRC cells

**FIGURE 3:**
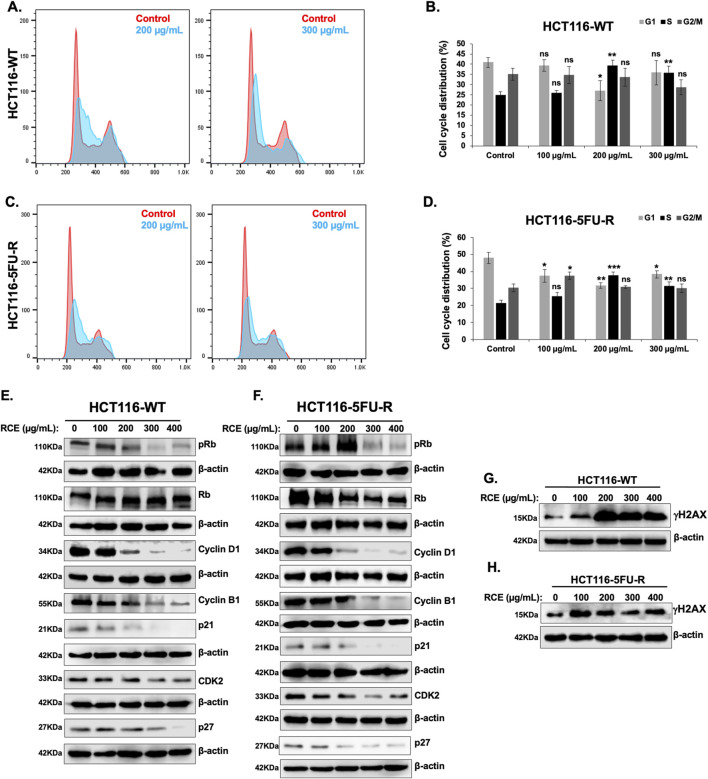
*Rhus coriaria* induces DNA-damage mediated S phase cell cycle arrest in parental and 5FU-resistant HCT-116 CRC cells **(A, B)** HCT-116-WT and **(C, D)** HCT-116-5FU-R cells were treated for 48 h with the indicated concentrations of RCE. The cells were then stained with the Muse^®^ Cell Cycle Kit and cell cycle distribution was analyzed with the MuseTM Cell Analyzer as described in the Materials and Methods section. Cell cycle profiles of control and 200 μg/mL RCE-treated **(A)** HCT-116-WT and **(C)** HCT-116-5FU-R cells. Cell cycle distribution of control and RCE-treated **(B)** HCT-116-WT and **(D)** HCT-116-5FU-R cells. Data is presented as the mean ± SEM; n = 4, in triplicate. ns: not significant, *p < 0.05, **p < 0.005, ***p < 0.001 (vs. 0 μg/mL); one-way ANOVA followed by Fisher’s LSD test. **(E, G)** HCT-116-WT and **(F, H)** HCT-116-5FU-R cells were treated for 48 h with the indicated concentrations of RCE, following which cells were collected for protein extraction. Western blot was performed to assess the expression of **(E, F)** phosphorylated (p-) Rb, total Rb, Cyclin D1, Cyclin B1, p21, p27, and CDK2, and **(G, H)** γH2AX.

To elucidate the mechanism(s) underlying the observed antiproliferative effects of RCE against parental and 5FU-resistant HCT-116 CRC cells, we analyzed its effect on cell cycle progression. To this end, we assessed the effect of 48 h of RCE treatment (0, 100, 200, and 300 μg/mL) on cell cycle distribution. We found that RCE significantly increased the S phase population of HCT-116-WT (Figures 3A, B) and HCT-116-5FU-R (Figures 3C, D) cells, starting at 200 μg/mL. This finding is in contrast to our previous report that RCE induced G1 arrest in breast and pancreatic cancer cells (El Hasasna et al., 2015; El Mahi et al., 2024) and can potentially be attributed to the molecular differences of different cancer types, as well as regulation of different molecular targets by RCE.

To confirm that RCE impairs cell cycle progression in parental and 5FU-resistant HCT-116 CRC cells and to clarify the molecular mechanisms underlying the same, we assessed the effect of RCE on the protein levels of various cell cycle markers. Rb is a key cell cycle regulator that is phosphorylated during G1, and whose phosphorylation is maintained across the other phases. Rb hypophosphorylation has been implicated in S phase arrest through regulation of S phase entry and progression (Knudsen et al., 2000). In fact, resveratrol (Wolter et al., 2001) and curcumin (Li et al., 2022), widely studied anti-cancer phytochemicals, have been reported to induce S phase arrest in CRC cells through Rb hypophosphorylation. Herein, we found that RCE treatment (48 h) decreased p-Rb levels without affecting total Rb levels in both HCT-116-WT (Figure 3E) and HCT-116-5FU-R (Figure 3F) cells. Consistent with previous literature on Rb-dependent S phase arrest (Knudsen et al., 2000) and our findings in TNBC cells (El Hasasna et al., 2015), RCE treatment (48 h) induced DNA damage as evidenced by increased γH2AX levels in both HCT-116-WT (Figure 3G) and HCT-116-5FU-R (Figure 3H) cells. However, it is important to note that the degree of DNA damage induction varied between HCT-116-WT (Figure 3G) and HCT-116-5FU-R (Figure 3H) cells. This is interesting as resistance to DNA damage has been implicated as a mechanism of 5FU resistance (Sethy and Kundu, 2021).

CDK2 is a cyclin-dependent kinase whose activation triggers Rb phosphorylation (Fisher, 2016), and it regulates G1/S transition and key events during S phase, such as initiation of DNA replication (Vermeulen et al., 2003). Consistent with p-Rb levels, RCE treatment (48 h) decreased CDK2 levels in both HCT-116-WT (Figure 3E) and HCT-116-5FU-R (Figure 3F) cells. This suggests that RCE modulated the Rb/CDK2 axis to induce S phase arrest in parental and 5FU-resistant HCT-116 CRC cells. Additionally, we assessed the levels of Cyclin D1 and Cyclin B1, which regulate entry into G1 phase and G2/M transition, respectively (Vermeulen et al., 2003). Consistent with our expectations, we found that RCE treatment (48 h) decreased the levels of both Cyclin D1 and Cyclin B1 in HCT-116-WT (Figure 3E) and HCT-116-5FU-R (Figure 3F) cells. This is consistent with previous literature which implicates cyclin D1 downregulation in S phase arrest, including in resveratrol-treated CRC cells (Wolter et al., 2001) and PM2.5-exposed lung carcinoma cells (Zhang et al., 2019). Furthermore, RCE induced perturbations in cell cycle progression were found to be independent of the cyclin-dependent kinase inhibitors p21 and p27 given the fact that the levels of these two proteins in both HCT-116-WT (Figure 3E) and HCT-116-5FU-R (Figure 3F) cells decreased with RCE treatment. Collectively, these findings confirm that RCE disrupts cell cycle progression at the S phase as an antiproliferative mechanism through induction of DNA damage in parental and 5FU-resistant HCT-116 CRC cells.

### 3.4 *Rhus coriaria* induces caspase-7-dependent apoptosis in HCT-116-WT CRC cells, while HCT-116-5FU-R cells are completely resistant to apoptosis

**FIGURE 4:**
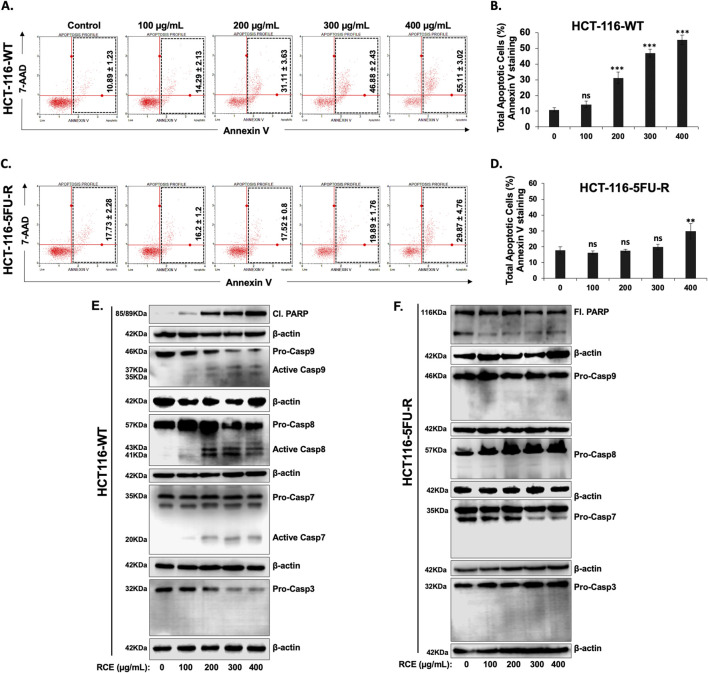
*Rhus coriaria* induces caspase-7-dependent apoptosis in parental HCT-116-WT CRC cells, but not in HCT-116-5FU-R CRC cells **(A, B)** HCT-116-WT and **(C, D)** HCT-116-5FU-R cells were treated for 48 h with the indicated concentrations of RCE, following which Annexin V-7-AAD staining was carried out using the Annexin V and Dead Cell kit (Millipore) and the events for total apoptotic cells were counted with the MuseTM Cell Analyzer as described in the Materials and Methods section. Data is presented as the mean ± SEM; n = 4. ns: not significant, **p < 0.005, ***p < 0.001 (vs. 0 μg/mL); one-way ANOVA followed by Fisher’s LSD test. **(E)** HCT-116-WT and **(F)** HCT-116-5FU-R cells were treated for 48 h with the indicated concentrations of RCE, following which cells were collected for protein extraction. Western blot was performed to assess the expression of cleaved and full length PARP, total and cleaved (activated) forms of Caspases 3, 7, 8, and 9.

We have previously reported that RCE induces apoptosis in HT-29 and Caco-2 CRC cells (Athamneh et al., 2017). Hence, we investigated the contribution of apoptosis in the observed anti-proliferative effect of RCE against parental and 5FU-resistant HCT-116 CRC cells. To this end, we performed Annexin/7-AAD staining following 48 h of RCE treatment. We found that starting at 200 μg/mL, RCE significantly increased the number of apoptotic cells in a concentration-dependent manner in HCT-116-WT cells (Figures 4A, B). Surprisingly however, RCE did not affect the number of total apoptotic cells in HCT-116-5FU-R cells, except at the highest concentration tested (400 μg/mL) (Figures 4C, D). Moreover, we also clarified that necroptosis did not contribute to RCE-mediated cell death in either HCT-116-WT or HCT-116-5FU-R cells. Indeed, necrostatin treatment did not have any effect on RCE-induced cell death in both cell lines (data not shown). These findings suggest that while apoptosis may be one of the mechanisms underlying RCE-mediated cell death in parental HCT-116-WT cells, apoptosis does not contribute to RCE-mediated cell death in HCT-116-5FU-R cells, which can perhaps be attributed to resistance to apoptosis in these cells.

To further clarify the contribution of apoptosis to RCE-mediated cell death in HCT-116-WT cells, we analyzed the expression of cleaved PARP, a classical marker of apoptosis, following 48 h of RCE treatment. As shown in Figure 4E, RCE induced a concentration-dependent increase in cleaved PARP levels in HCT-116-WT cells, in line with the results of Annexin/7-AAD staining (Figure 4A). Next, we sought to confirm the mechanism underlying the observed induction of apoptosis. To this end, we assessed the activation of initiator caspases −8 and −9, and executioner caspases −3 and −7 in RCE-treated HCT-116-WT cells. As shown in Figure 4E, we found that in HCT-116-WT cells, RCE activated both caspases-8 and -9, which are initiator caspases of the extrinsic and intrinsic pathways of apoptosis, respectively, and activated the executioner caspase-7. Noteworthy, we observed a decrease in pro-caspase-3 levels, which was not accompanied by an increase in cleaved caspase-3 levels in HCT-116-WT cells (Figure 4E). This is in line with our previous report in HT-29 and Caco-2 CRC cells that RCE induces proteasome-mediated degradation of pro-caspase-3 (Athamneh et al., 2017). Collectively, these findings suggest that RCE induces caspase-7-dependent apoptosis in parental HCT-116-WT cells through involvement of both the intrinsic and extrinsic pathways. Further, DNA damage (Figure 3G) induced by RCE in HCT-116-WT cells could be an upstream inducer of the intrinsic pathway of apoptosis. In stark contrast to the results with HCT-116-WT cells, PARP cleavage was not noted in HCT-116-5FU-R cells, which prompted us to use an antibody targeting both full length and cleaved PARP, which were both found to be unaffected by RCE treatment (48 h) (Figure 4F). Consistent with the absence of PARP cleavage, the activation of canonical caspases −8, −9, −7 and −3 was not noted in HCT-116-5FU-R cells (Figure 4F), even at very high exposure. These findings are line with the results of the Annexin V/7-AAD staining (Figures 4C, D), suggesting that HCT-116-5FU-R cells are resistant to RCE-mediated apoptosis. Additionally, it suggests that apoptosis does not account for RCE-induced cell death in HCT-116-5FU-R cells.

**FIGURE 5:**
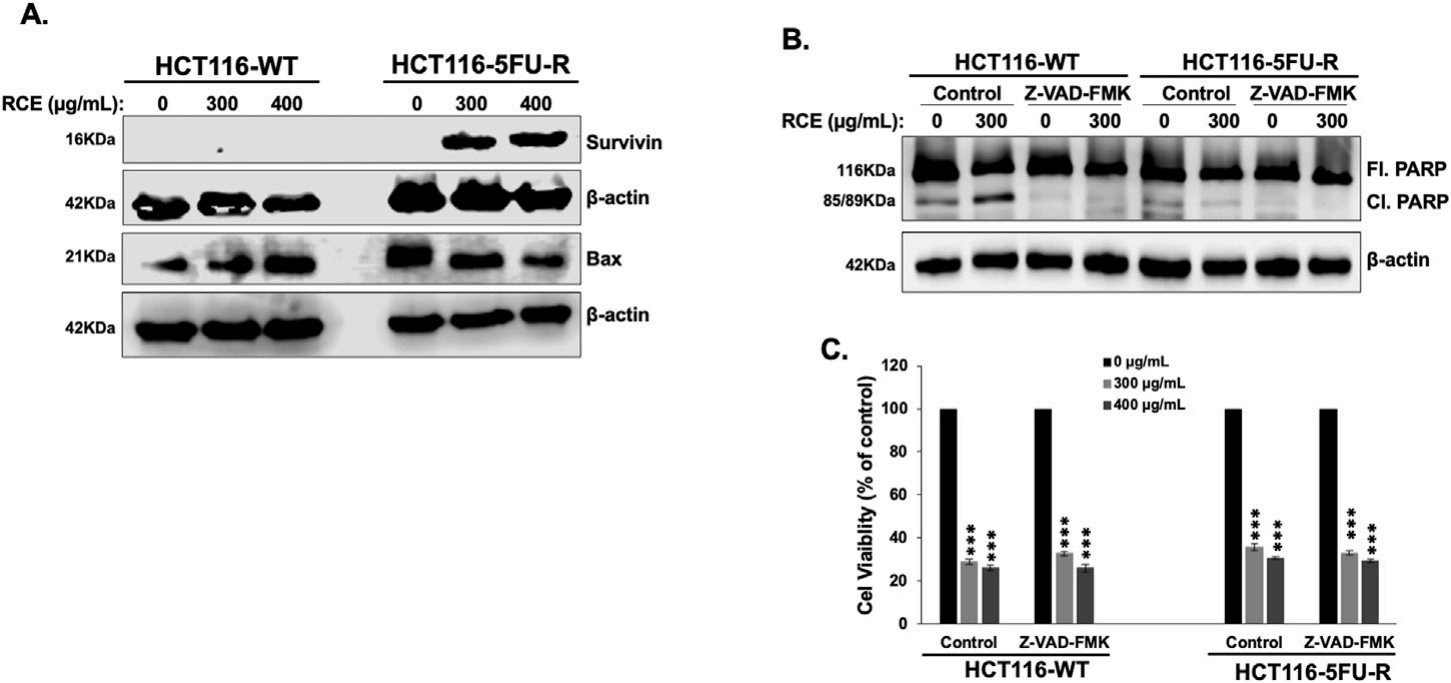
*Rhus coriaria* induces caspase-dependent PARP cleavage in parental HCT-116-WT CRC cells and Survivin expression in HCT-116-5FU-R CRC cells. **(A)** HCT-116-WT and HCT-116-5FU-R cells were treated for 48 h with the indicated concentrations of RCE, following which cells were collected for protein extraction. Western blot was performed to assess the expression of Survivin. **(B)** HCT-116-WT and HCT-116-5FU-R cells were treated for 48 h with 300 μg/mL RCE with or without pretreatment for 2 h with 50 µM Z-VAD-FMAK (a pan-caspase inhibitor), following which cells were collected for protein extraction. Western blot was performed to assess the expression of full length (Fl.) and cleaved (Cl.) PARP. **(C)** HCT-116-WT and HCT-116-5FU-R cells were treated with 300 and 400 μg/mL of RCE for 48 h, with or without pretreatment for 2 h with 50 µM Z-VAD-FMAK (a pan caspase inhibitor). Following treatment, cell viability was assessed using MTT assay. Data is presented as the mean ± SEM; n = 4, in triplicate. ns: not significant, ***p < 0.001; one way ANOVA followed by Fisher’s LSD test.

To clarify a possible mechanism underlying the observed resistance to apoptosis in HCT-116-5FU-R cells, we assessed the expression of Survivin, a member of the inhibitor of apoptosis protein family, which inhibits the activity of caspases −3, −7, and −9 (Tamm et al., 1998), and that of Bax, a member of the Bcl-2 family, which regulates the intrinsic pathway of apoptosis (Peña‐Blanco and García‐Sáez, 2018). We found that 48 h of RCE treatment (at 300 and 400 μg/mL) induced the expression of Survivin and downregulated the expression of Bax in HCT-116-5FU-R cells. In HCT116-WT cells, RCE had no effect on survivin expression while it upregulated Bax (Figure 5A). This dichotomy is also supported by the difference in RCE-mediated DNA damage between HCT-116-WT (Figure 3G) and HCT-116-5FU-R (Figure 3H) cells, given that DNA damage is an important trigger of apoptosis (Roos and Kaina, 2006). Hence, we propose that resistance to DNA damage, Survivin induction, and downregulation of Bax are possible mechanisms underlying resistance to apoptosis in HCT-116-5FU-R cells. We also clarified the role of caspases in RCE-mediated PARP cleavage in HCT-116-WT cells as PARP is a substrate of various proteases apart from caspases (Chaitanya et al., 2010). To this end, we pretreated HCT-116-WT and HCT-116-5FU-R cells with the pan-caspase inhibitor Z-VAD-FMK 2 h prior to RCE treatment (48 h) and assessed the protein levels of full length and cleaved PARP. We found that pretreatment with Z-VAD-FMK reversed RCE-mediated accumulation of cleaved PARP in HCT-116-WT cells (Figure 5B), further clarifying the role of caspases in RCE-mediated apoptosis in the parental HCT-116 cells. Noteworthily, accumulation of cleaved PARP was not noted in HCT-116-5FU-R cells in this experiment (Figure 5B), consistent with our findings in Figure 4F. Given these surprising results, and our previous reports of minimal contribution of apoptosis to RCE-mediated cell death in TNBC (El Hasasna et al., 2015) and CRC (Athamneh et al., 2017) cells, we used the pan-caspase inhibitor Z-VAD-FMK to clarify whether apoptosis is the main mechanism underlying RCE-mediated cell death in HCT-116-WT cells. As expected, caspase inhibition with Z-VAD-FMK did not have an effect on the viability of RCE-treated HCT-116-5FU-R cells. However, surprisingly, Z-VAD-FMK pretreatment did not rescue HCT-116-WT cells from RCE-mediated cell death (Figure 5C). These findings suggest that while RCE induces apoptosis in HCT-116-WT cells, it is not the primary mechanism of RCE-mediated cell death in parental HCT-116-WT cells.

### 3.5 *Rhus coriaria* induces non-canonical Beclin-1-independent autophagy in parental and 5FU-resistant HCT-116 CRC cells

**FIGURE 6:**
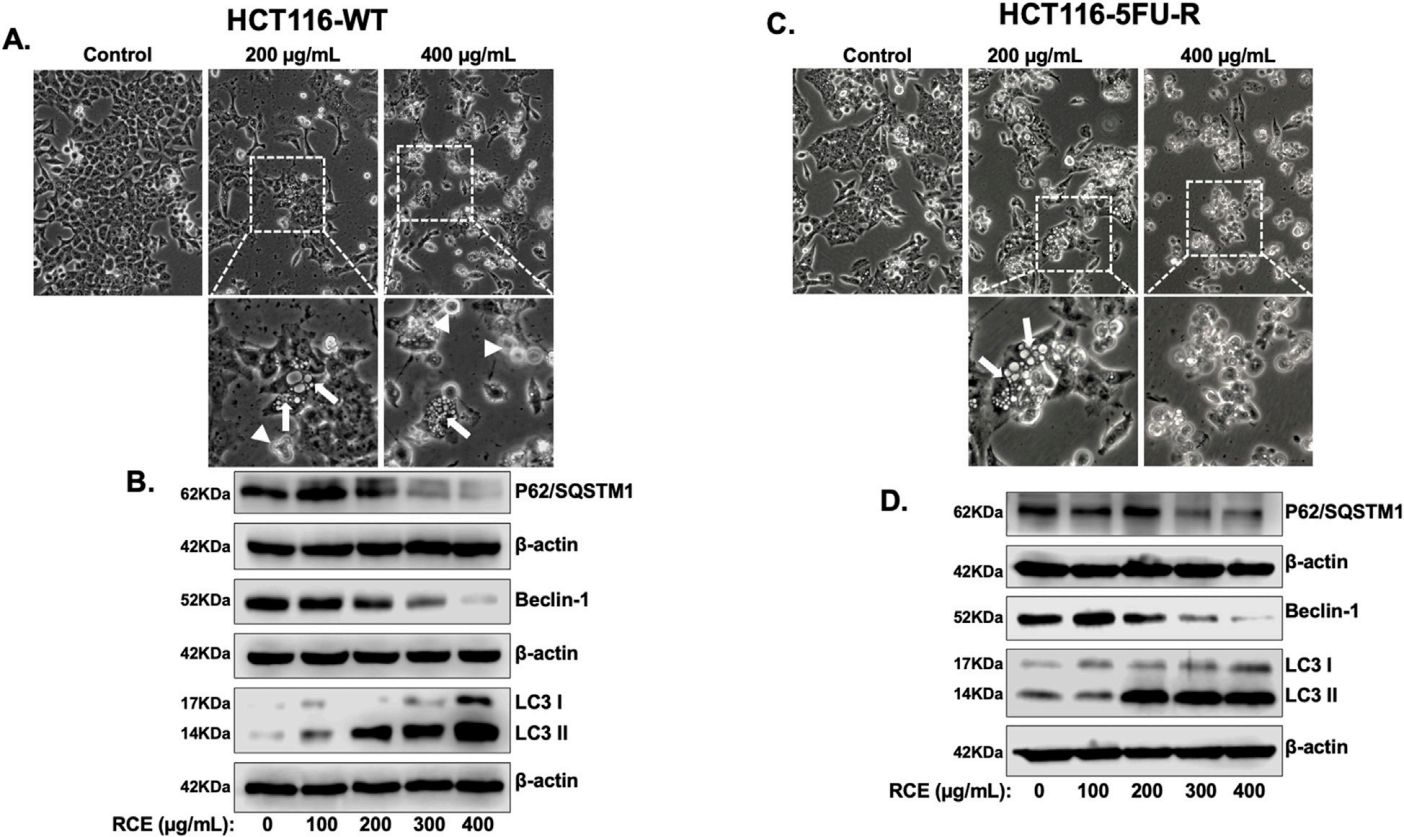
*Rhus coriaria* induces Beclin 1-independent autophagy in parental and 5FU-resistant HCT-116 CRC cells. HCT-116-WT and HCT-116-cells were treated for 48 h with the indicated concentrations of RCE. **(A, B)** Morphological changes induced in RCE-treated **(C)** HCT-116-WT and **(D)** HCT-116-5FU-R cells were observed by light microscopy (EVOS XL Core Cell Imaging System; Life Technologies, Waltham, MA, United States); 40×. Representative images are shown. White boxes show zoom in areas with cytoplasmic vacuolation (arrows) and/or dying cells (arrowheads). **(C, D)** RCE-treated **(C)** HCT-116-WT and **(D)** HCT-116-5FU-R were collected for protein extraction. Western blot was performed to assess the expression of LC3, p62, and Beclin-1.

Given that apoptosis did not contribute to RCE-mediated cell death in HCT-116-5FU-R cells, and apoptosis inhibition did not rescue HCT-116-WT cells from the same, we explored other forms of cell death, namely, autophagy. Previously, we reported that RCE induces autophagic cell death in HT-29 and Caco-2 CRC cells (Athamneh et al., 2017). Consistently, in the present study, morphological examination following RCE treatment (48 h) revealed massive cytoplasmic vacuolation (arrows), indicative of autophagy, in HCT-116-WT (Figure 6A) and HCT-116-5FU-R cells (Figure 6C). To confirm the induction of autophagy, we examined the protein levels of three integral autophagy markers, LC3-II, sequestome 1 (p62/SQSTM1), and Beclin-1. The formation of autophagosomes is characterized by the conversion of cytosolic LC3-I to its lipid-conjugated and membrane-bound form LC3-II (Klionsky et al., 2021). We found that RCE treatment (48 h) induced a concentration-dependent increase in LC3-II accumulation in both HCT-116-WT (Figure 6B) and HCT-116-5FU-R (Figure 6D) cells. This was concomitant with a decrease in p62 levels in HCT-116-WT (Figure 6C) and HCT-116-5FU-R (Figure 6D) cells. p62 is an autophagy receptor that facilitates the recruitment of ubiquitinated proteins to the growing phagophore, whose degradation is an indicator of autophagic flux (lysosome-mediated degradation of autophagy cargo) (Klionsky et al., 2021). Collectively, these findings suggest that RCE increases autophagic flux in parental and 5FU-resistant HCT-116 CRC cells. Furthermore, the induction of autophagy was found to be independent of Beclin-1, which is part of the phagophore nucleation class III PI3K complex involved in autophagosome formation (Klionsky et al., 2021), as evidenced by the concentration-dependent decrease in Beclin-1 levels in HCT-116-WT (Figure 6B) and HCT-116-5FU-R (Figure 6D) cells. This is consistent with our previous findings in HT-29 and Caco-2 CRC cells (Athamneh et al., 2017). Collectively, these findings suggest that RCE induces Beclin-1-independent autophagy in parental and 5FU-resistant HCT-116 CRC cells.

### 3.6 *Rhus coriaria* inhibits the mTOR-STAT3 and p38MAPK pathways to induce autophagy in parental and 5FU-resistant HCT-116 CRC cells

**FIGURE 7:**
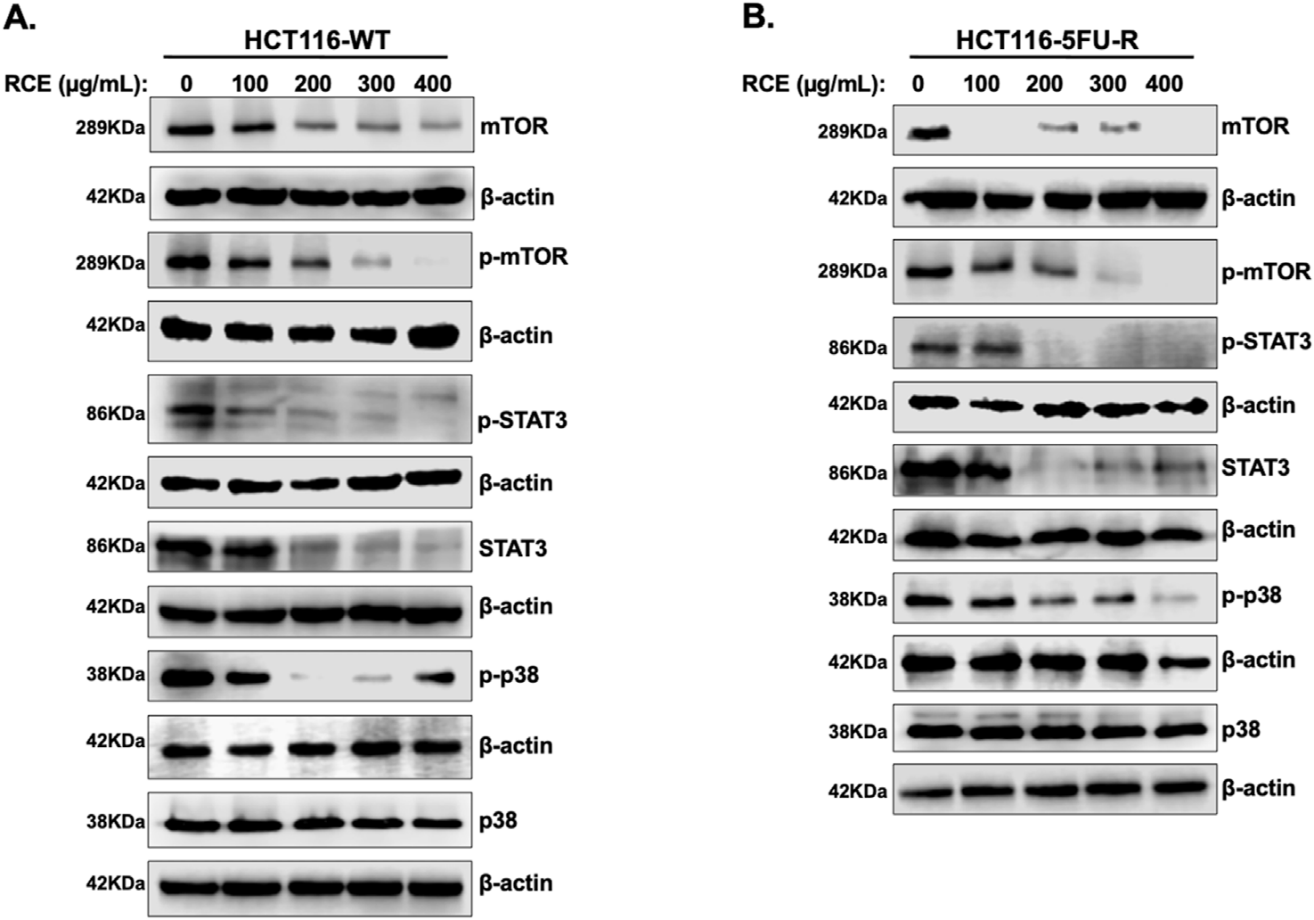
*Rhus coriaria* inhibits the mTOR-STAT3 and p38MAPRK pathways in parental and 5FU-resistant HCT-116 CRC cells. **(A)** HCT-116-WT and **(B)** HCT-116-5FU-R cells were treated for 48 h with the indicated concentrations of RCE, following which cells were collected for protein extraction. Western blot was performed to assess the expression of phosphorylated and total forms of mTOR, STAT3 and p38MAPRK.

Next, we examined the mechanism(s) underlying RCE-mediated induction of Beclin 1-independent autophagy in parental and 5FU-resistant HCT-116 CRC cells. Previously, we reported that RCE induces autophagy through proteasomal-mediated degradation of autophagy regulator mTOR1 in HT-29 and Caco-2 CRC cells (Athamneh et al., 2017). Under normal physiological conditions, mTOR1 directly inhibits autophagy by inhibiting the ULK1 complex, which is involved in autophagy induction (Kma and Baruah, 2022). Hence, we analyzed the levels of total and p-mTOR1 by Western blot analysis. We found that RCE treatment (48 h) led to a concentration-dependent decrease in p-mTOR (Ser2448) and total mTOR levels in both HCT-116-WT (Figure 7A) and HCT-116-5FU-R (Figure 7B) cells. We also examined the effect of RCE treatment (48 h) on the protein levels of other proteins reported to regulate autophagy, namely, STAT3 and p38MAPK. Herein, we found that RCE treatment (48 h) led to a concentration-dependent decrease in p-STAT3 (Tyr705) and total STAT3 levels in both HCT-116-WT (Figure 7A) and HCT-116-5FU-R (Figure 7B) cells, consistent with our previous findings in TNBC cells (El Hasasna et al., 2016). We previously reported that RCE activates the p38 pathway to induce autophagy in TNBC cells (El Hasasna et al., 2015); hence, we explored the effect of RCE on the same in parental and 5FU-resistant HCT-116 CRC cells. Surprisingly, we found that in contrast to our previous findings (El Hasasna et al., 2015), RCE decreased the phosphorylation of p38MAPK (Thr180/Tyr182) without affecting total p38MAPK levels in both HCT-116-WT (Figure 7A) and HCT-116-5FU-R (Figure 7B) cells. These findings are not unsurprising in the broader context of cancer as the p38MAPK pathway plays dual roles in autophagy (He et al., 2018; Jiang et al., 2014). Collectively, these findings suggest that inhibition of the mTOR, STAT3 and p38MAPK pathways might be involved, at least in part, in RCE-induced autophagy in parental and 5FU-resistant HCT-116 CRC cells.

### 3.7 *Rhus coriaria*-induced cell death is dependent on autophagy and the proteasome pathway in parental and 5FU-resistant HCT-116 CRC cells

**FIGURE 8:**
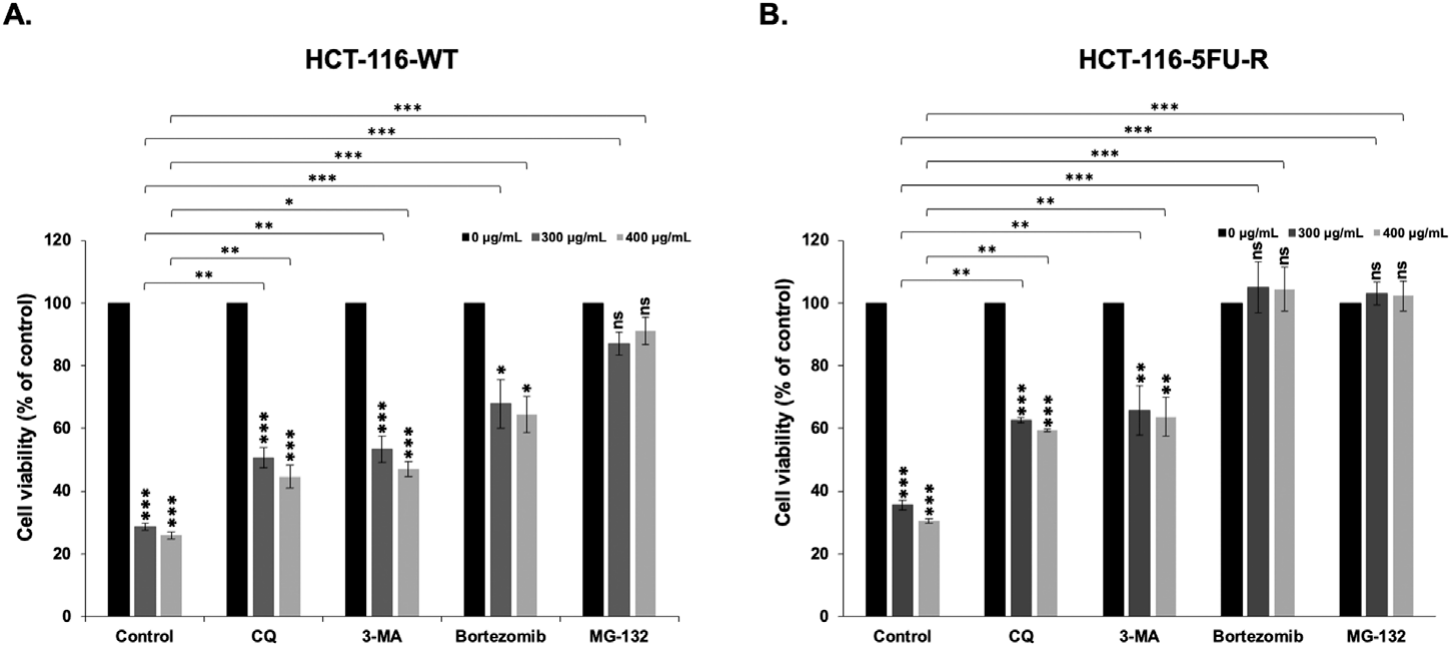
*Rhus coriaria*-induced cell death is dependent on the proteasome pathway and autophagy in parental and 5FU-resistant HCT-116 CRC cells. **(A)** HCT-116-WT and **(B)** HCT-116-5FU-R cells were treated with 300 and 400 μg/mL of RCE for 48 h, with or without pretreatment for 2 h with autophagy inhibitors (50 µM CQ and 5 mM 3-MA) and proteasome inhibitors (15 nM Bortezomib and 15 µM MG-132). Following treatment, cell viability was assessed using MTT assay. Data is presented as the mean ± SEM; n = 4–5, in triplicate. ns: not significant, *p < 0.05, **p < 0.005, ***p < 0.001; one-way ANOVA followed by Fisher’s LSD test. Asterisks on top of the bars denote significance compared to 0 μg/mL within the treatment group, whereas connecting brackets denote significance compared to the corresponding concentration of the control group.

Given that we clarified that apoptosis is not the main mechanism of RCE-mediated cell death in parental and 5FU-resistant HCT-116 CRC cells, we explored the contribution of other pathways. Autophagy is known to induce programmed cell death type II; hence, we pretreated parental and 5FU-resistant HCT-116 CRC cells with the autophagy inhibitors CQ (50 µM) and 3 MA (5 mM) for 2 h prior to RCE treatment (48 h). In both HCT-116-WT (Figure 8A) and HCT-116-5FU-R (Figure 8B) cells, pretreatment with either autophagy inhibitor 3-MA (a type III phosphatidylinositol 3 kinase inhibitor) or CQ (an inhibitor of autophagosome–lysosome fusion) partially rescued the viability of RCE treated HCT-116-WT and HCT-116-5FU-R cells. This finding is in line with our previous report in HT-29 CRC cells (Athamneh et al., 2017). This finding suggests that autophagy contributes, at least in part, to RCE-mediated cell death in parental and 5FU-resistant HCT-116 CRC cells. We also explored the contribution of the proteasome pathway given our previous findings in HT-29 CRC cells that RCE targets specific proteins to proteasome-mediated degradation (Athamneh et al., 2017). To this end, we pretreated parental and 5FU-resistant HCT-116 CRC cells with the proteasome inhibitors bortezomib and MG-132 for 2 h before RCE treatment (48 h). Interestingly, pretreatment with the proteasome inhibitors bortezomib and MG-132 completely rescued HCT-116-5FU-R cells from RCE-induced cell death (Figure 8B), and markedly rescued HCT-116-WT cells from the same (Figure 8A). This finding is in line with our previous report where pretreatment with MG-132 significantly improved the viability of RCE-treated HT-29 CRC cells (Athamneh et al., 2017). Further, these findings suggest that proteasome-mediated degradation of key regulators proteins may underlie RCE-mediated cell death in parental and 5FU-resistant HCT-116 CRC cells.

### 3.8 *Rhus coriaria* inhibits the growth of human pancreatic cancer in a chick embryo xenograft model

**FIGURE 9:**
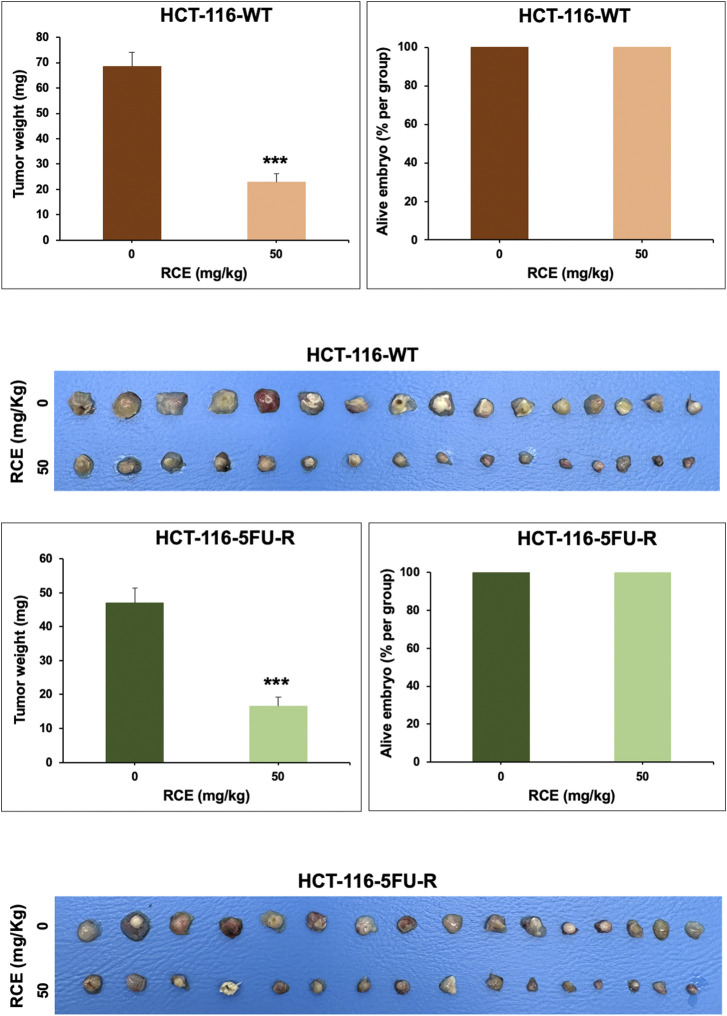
*Rhus coriaria* extract inhibits the growth of parental and 5FU-resistant HCT-116 CRC cells in a chick embryo xenograft model **(A)** HCT-116 and **(B)** HCT-116-5FU-R cells were inoculated on the chorioallantoic membrane (CAM) of chick embryos on embryonic day 9 (E9). Starting on E11, tumors were treated every 48 h with 50 mg/kg of RCE as described in the Materials and Methods section. On E17, tumors were collected and weighted. Tumor weight (mg) in control- and RCE-treated chick embryos and the number of surviving control- and RCE-treated chick embryos were quantified. Data is presented as the mean ± SEM; n = 16/group. ***p < 0.001; The unpaired t-test wasapplied to evaluate the difference between the two groups.

Lastly, we confirmed our *in vitro* anticancer effects using an *in ovo* chick embryo xenograft model. To this end, HCT-116-WT (Figure 9A) and HCT-116-5FU-R (Figure 9B) CRC cells were xenografted on the CAM of fertilized chicken eggs and the formed tumors were treated with the vehicle (ethanol) or 50 mg/kg of RCE. Firstly, there was no difference in the number of surviving control and RCE-treated embryos (Figure 9), confirming that RCE does not exhibit cytotoxicity *in vivo*. We found that 50 mg/kg of RCE significantly inhibited tumor growth by 66.6% and 64.6% for HCT-116-WT (Figure 9A) and HCT-116-5FU-R (Figure 9B) CRC cells, respectively, compared with their respective controls. Collectively, these findings confirm the anticancer activity of RCE against parental and 5FU-resistant HCT-116 CRC cells *in ovo*.

## 4 Discussion

Natural products, specifically plant-derived compounds, are promising cancer therapeutic options. In fact, of the 185 small molecules developed as anticancer drugs between 1981 and 2019, 33.5% were natural products or their derivatives (Newman and Cragg, 2020). The identification of these natural products starts with the investigation of plant extracts with potent biological effects. Consistently, we have previously reported on the anti-cancer effects of RCE against TNBC (El Hasasna et al., 2015; El Hasasna et al., 2016) and CRC (Athamneh et al., 2017) cells. Similarly, other groups have also reported on the anti-cancer effect of RCE against breast cancer (Gabr S and Alghadir A, 2021; Ghorbani et al., 2018; Kubatka et al., 2020). In the present study, we investigated the anti-cancer effect of RCE against 5FU-resistant CRC cells. Our findings demonstrate that RCE inhibits the viability and colony formation and growth ability of parental and 5FU-resistant HCT-116 CRC cells. Mechanistically, we found that RCE induces DNA damage-mediated S phase arrest, and non-canonical Beclin-1-independent autophagy in parental and 5FU-resistant CRC cells through inhibition of the mTOR-STAT3 and p38MAPK pathways. With respect to apoptosis, while RCE induced caspase-7-dependent apoptosis in parental HCT-116-WT cells, HCT-116-5FU-R cells were resistant to RCE-mediated apoptosis possibly through resistance to DNA damage, induction of Survivin, expression and downregulation of Bax. Additionally, we clarified that autophagy and the proteasome pathway contributed to RCE-mediated cell death in parental and 5FU-resistant HCT-116 CRC cells, with the proteasome pathway most likely being the driving force behind RCE-mediated cell death in both cell lines through degradation of key molecular targets. Lastly, we confirmed that RCE inhibited the growth of parental and 5FU-resistant HCT-116 xenografts in a chicken embryo xenograft model.

Apoptosis (PCD-I), autophagy (PCD-II), necroptosis, and ferroptosis are the main mechanisms of programmed cell death in cancer cells (Dixon et al., 2012; Kist and Vucic, 2021). Herein, we showed that *Rhus coriaria* extract induced cell death in parental and 5FU-resistant HCT-116 CRC cells. Ferroptosis and necroptosis were not induced in RCE-treated cells as inhibition by N-acetylcysteine (ferroptosis) or necrostatin (necroptosis) did not abolish the cytotoxic effects of RCE in both cell lines (data not shown). Rather, we found that in the parental HCT-116-WT CRC cells, RCE induced Beclin-1 independent autophagy and activated both extrinsic and intrinsic apoptotic pathways. This agrees with our previous data on HT-29 and Caco-2 CRC cells (Athamneh et al., 2017). However, only autophagy was induced by RCE in the 5FU-resistant CRC (HCT-116-5FU-R) cells; apoptosis was not induced by RCE in HCT-116-5FU-R cells. Indeed, no active caspases (effectors and executioners) nor PARP cleavage could be detected in these cells. This is in line with previous data documenting that 5FU-resistant CRC cells acquire resistance to apoptosis (Sousa-Squiavinato et al., 2022). Resistance/evasion to apoptosis can be achieved through different mechanisms, such as the inhibition of proapoptotic proteins, overexpression of antiapoptotic proteins, and the inhibition of the apoptotic machinery (Azwar et al., 2021). Survivin, an inhibitor of apoptosis, was reported to inhibit both extrinsic and intrinsic apoptotic pathways through caspase-dependent and -independent mechanisms (Warrier et al., 2020). Similarly, downregulation of Bax, a pro-apoptotic molecule, was reported to contribute to CRC pathogenesis and 5FU resistance (Manoochehri et al., 2014). Interestingly, we found that RCE-treated HCT-116-5FU-R expressed high protein levels of Survivin and low protein levels of Bax. Hence, it is legitimate to suggest that induction of Survivin expression and downregulation of Bax expression account for the inhibition of apoptosis in 5FU-resistant HCT-116 CRC cells.

Based on our data, we can conclude that PCD-II is the main mechanism of cell death induced by RCE in both cell lines most probably because of excessive autophagy. Apoptosis might be activated in parental HCT-116-WT cells as secondary mechanism of RCE-induced cell death, likely as a consequence of excessive cellular damage induced by longer exposure of the cells to RCE. This claim is supported by the following lines of evidence: (i) massive cell death occurred in parental HCT-116-WT cells even when apoptosis was inhibited by Z-VAD-FMK (a pan-caspase inhibitor); (ii) partial abrogation of RCE-induced cell death was observed in parental and 5FU-resistant CRC cells when autophagy was chemically inhibited by either CQ or 3-MA. This is in agreement with previously published data on MDA-MB-231 TNBC (El Hasasna et al., 2015) and HT-29 and Caco-2 CRC (Athamneh et al., 2017) cancer cells. Moreover, (iii) cell death occurred in the 5FU-resistant CRC (HCT-116-5FU-R) cells in the absence of activation of PCD-I. Further, supporting the idea that autophagy is the main mechanism of RCE-induced cell death, it has been reported that non-canonical (Beclin-1-independent) autophagy, as was observed in our study with RCE, is associated with PCDII (Scarlatti et al., 2008; Shrivastava et al., 2011).

It has been largely reported that the inhibition of mTORC1 promotes autophagy, while its upregulation inhibits autophagy (Codogno and Meijer, 2005). Indeed, rapamycin, an allosteric inhibitor of mTOR1, is the most commonly used inducer of autophagy (Klionsky et al., 2021). We have previously reported that *Rhus coriaria*-induced autophagy was a consequence of the inhibition of mTOR pathway in HT-29 and Caco-2 CRC cells (Athamneh et al., 2017) through targeted proteasomal degradation of mTOR (Athamneh et al., 2017). In line with these findings, herein, we showed that at concentrations that induce autophagy (i.e., 200, 300 and 400 μg/mL), RCE dramatically reduced the protein levels of both phosphorylated- and total mTOR in parental as well as 5FU-resistant cells. Hence, we suggest that the inhibition of mTOR pathway, most probably due to proteasomal degradation of mTOR1 protein, may at least in part, account for the observed induction of autophagy. It is noteworthy to mention that studies have shown that the inhibition of the mTOR pathway can not only induce autophagy, but also stimulate protein degradation (Klionsky and Emr, 2000; Zhao et al., 2015).

Nuclear and cytoplasmic STAT3 is known to negatively regulate autophagy through transcriptional regulation of autophagy-associated genes, disruption of lysosomal fusion, upregulation of microRNAs that target autophagy-related genes (such as ULK1), sequestration of FOXO proteins (transcription factors that regulate expression of autophagy-related genes), among other mechanisms (Xu et al., 2022). Further, STAT3 regulates various other aspects of cancer cells, including migration and metastasis (Tolomeo and Cascio, 2021). Metformin was shown to induce autophagy through the inactivation of the STAT3 and mTOR pathways in esophageal squamous cell carcinoma (Feng et al., 2014). Further, siRNA-mediated knockdown of STAT3 was sufficient to induce autophagy in these cells (Feng et al., 2014). Similarly, carnosol, a natural polyphenolic compound, was also shown to induce Beclin-1-independent autophagy through inactivation of the STAT3 and mTOR pathways (Alsamri et al., 2022). Wang et al. reported that cross talk between the STAT3 and mTOR signaling pathways plays a role in sensitivity to chemotherapy in osteosarcoma cells (Wang et al., 2020). Indeed, they showed that the chemical inhibition of STAT3 activation by napabucasin also resulted in the inhibition of activation of mTOR (Wang et al., 2020). On the other hand, rapamycin, an inhibitor of mTOR, also decreased the levels of p-STAT3 (Wang et al., 2020). In line with the latter finding, several studies reported that STAT3 is a direct substrate of mTOR, which phosphorylates STAT3 at tyrosine 705 and serine 727 (Yokogami et al., 2000; Dodd et al., 2015; Bezzerri et al., 2016). Herein, we showed that RCE inhibited both the STAT3 and mTOR pathways, most probably through downregulation of their respective protein levels in both parental and 5FU-resistant CRC cell lines. RCE was also shown to induce Beclin-1-independent autophagy in TNBC cells. Interestingly, the STAT3 pathway was also inhibited in RCE-treated TNBC cells (El Hasasna et al., 2016). Thus, altogether, our data suggest that, in addition to mTOR inhibition, STAT3 inhibition might also implicated in RCE-induced autophagy in CRC cells. Further investigations are needed to establish the individual role of these two pathways in the induction of RCE-mediated autophagy.

We have previously reported that *Rhus coriaria* induced autophagic cell death in HT-29 and Caco-2 CRC cell lines by targeting several key proteins involved in autophagy inhibition (mTOR and AKT) to proteasomal degradation (Athamneh et al., 2017). This correlated with an increase in the overall level of protein ubiquitination and stimulation of the ubiquitin proteasome system (UPS) (Athamneh et al., 2017). The inhibition of proteasome activity by the proteasome inhibitor MG-132 not only blocked RCE-induced autophagy, but also significantly reduced RCE-induced cell death (Athamneh et al., 2017). Similar to these findings, herein, we found that RCE dramatically reduced the protein levels of mTOR, probably by targeting it to proteasomal degradation. Importantly, we showed that inhibition of the UPS using the proteasome inhibitors MG-132 and bortezomib restored the cellular viability of RCE-treated cells to a level comparable to the controls in both parental and 5FU-resistant CRC cell lines, thus highlighting the role of proteasome activity in RCE-mediated effects in these cells. Paclitaxel, a microtubule stabilizing agent, was reported to induce cell death in many cancer types through induction of mitotic catastrophe, autophagy, and apoptosis (Khing et al., 2021). Recently, Hu et al. showed that inhibition of the proteasome by MG-132 or bortezomib reduced paclitaxel-induced cell death in 5–8F and 6–10B nasopharyngeal cancer cells (Hu et al., 2021).

It is well documented that p38 MAPK is implicated directly or indirectly in many cellular responses to multiple stresses which includes cell cycle arrest, apoptosis and autophagy (Han et al., 2020; Canovas and Nebreda, 2021). Several studies showed that inhibition of p38 MAPK induces autophagy in colon cancer cells. In fact, triterpenes from the medicinal mushroom *Ganoderma lucidum* induced autophagic cell death (PCD-II) in HT-29 CRC cells through inhibition of p38 MAPK (Thyagarajan et al., 2010). Additionally, chemical inhibition of p38 MAPK using PH797804 (a p38 specific inhibitor) reduced growth of patient-derived xenografts (PDX) derived from CRC tumors (Gupta et al., 2015). Interestingly, PH797804-treated PDX showed reduced levels of phosphorylated STAT3 compared to non-treated PDX (Gupta et al., 2015); however, total STAT3 protein levels were not determined. In the present study, we found that RCE induced a decrease in the protein levels of phosphorylated p38 MAPK without affecting total p38 MAPK protein levels, hence suggesting that inhibition of p38 MAPK could also contribute to RCE-induced autophagy. Recently, Leestemaker et al. linked the p38 MAPK pathway to the ubiquitin proteasome system. Indeed, they showed that the chemical inhibition or genetic depletion of p38 MAPK enhanced the proteasome activity in MelJuSo human melanoma cells (Leestemaker et al., 2017). Herein, we showed that RCE inhibited the p38 MAPK pathway in both parental and 5FU-resistant CRC cells. It is thus tempting to speculate that p38 inhibition by RCE contributes, at least in part, to the activation of the UPS leading to the degradation of mTOR and STAT3, which consequently induces autophagy. Further investigations are needed to clarify the exact role of p38 MAPK inhibition in RCE-mediated effects.

In the present study, we showed that RCE induces DNA damage in both parental and 5FU-resistant CRC cell lines. However, the level of DNA damage induction varied between the two cell lines; HCT-116-5FU-R exhibited less DNA damage in response to RCE treatment compared to the parental cell line. One possible explanation for this variation is potential resistance to DNA damage in 5FU-resistant cells. Indeed, resistance to DNA damage has been implicated as a mechanism of 5FU resistance through extensive DNA repair potentiality (Sethy and Kundu, 2021). Noteworthily, while this can decrease induction of DNA damage, it does not completely abolish it and nor does it abolish the cellular response to DNA damage. Hence, we hypothesize that HCT-116-5FU-R cells may have possibly acquired resistance to DNA damage, accounting for the observed differences. Nonetheless, we cannot rule out the involvement of other mechanism(s). It is well known that various genotoxic drugs (such as cisplatin, olaparib, resveratrol, curcumin, etc.) mediate their anticancer effect(s) through induction of DNA damage and consequently accumulation of γH2AX (Prabhu et al., 2024). In most cases, the accumulation of DNA damage, a primary response to drug exposure, leads to the activation of apoptotic pathways, which can further potentiate DNA damage as a consequence of apoptotic DNA fragmentation. In fact, H2AX phosphorylation is critical for DNA degradation mediated by caspase-activated DNase (CAD) activity during apoptosis (Lu et al., 2006) and the inhibition of apoptosis by the pan-caspase inhibitor Z-VAD-FMK inhibits γH2AX formation (Chiu et al., 2008). We have previously shown that DNA damage is the earliest response to RCE treatment in TNBC cells (El Hasasna et al., 2015), preceding autophagy, which in turn preceded apoptosis. Herein, we show that RCE induces DNA damage in both wild-type and 5FU-resistant CRC cells. Hence, we hypothesize that in both cell lines, DNA damage is likely the earliest response to RCE treatment, which serves as a trigger for subsequent cellular responses, i.e., autophagy followed by apoptosis. With respect to accumulation of DNA damage secondary to apoptosis (as a consequence of DNA fragmentation), in HCT-116-WT cells, higher levels of DNA damage were observed at RCE concentrations that triggered pronounced apoptosis (200 μg/mL and above; Figures 3G, 4A). Conversely, in HCT-116-5FU-R cells, which are resistant to apoptosis, no further accumulation of DNA damage was noted with increasing concentrations of RCE (Figures 3H, 4B). Further investigations are needed to elucidate the exact mechanisms that contribute to resistance to DNA damage in HCT-116-5FU-R cells.

The anticancer activity of RCE can be attributed to the diversity of its phytochemical constituents. Over 200 phytochemicals have been characterized in *Rhus coriaria* (Alsamri et al., 2021). In the present study, using HPLC-MS approach, we identified 8 phytochemicals in RCE that may potentially account for the observed effects. Of these, the anticancer activity of 1-O-(4-Coumaroyl)-beta-D-glucose, digallic acid, sespendole, and phloretin 2′-glucoside has not been elucidated to date, representing a future direction of research. While the anticancer activity of quininic acid has not been explored, it has been implicated in the anticancer effects of various plants, including *Eugenia uniflora* (G De Oliveira et al., 2023), peanut skin (*Arachis hypogea*) (Cordeiro-Massironi et al., 2023), among others. Genistin was reported to exert anticancer effects against breast (MCF7 and MDA-MB-231) (Hwang et al., 2020) and glioma (Lan et al., 2024) cells; however, its anticancer activity has not been explored in CRC. The other two identified phytochemicals, gallic acid and quercetin are well explored for their anticancer effects, including in CRC. In fact, quercetin has been reported to reverse 5FU resistance in CRC cells (HCT-116) by modulating the Nrf2-HO-1 pathway (Tang et al., 2023). Similarly, gallic acid has been reported to downregulate STAT3 and AKT pathways in CRC cells (HCT-116 and HT-29) (Lin et al., 2021). These two phytochemicals hence represent promising avenues for research. Consistently, we are currently working on identifying the bioactive fractions of RCE to further narrow down its active constituents, to develop a novel anti-cancer agent, specifically for 5FU-resistant CRC.

In conclusion, this report not only confirms our previous findings that *Rhus coriaria* exhibits anti-TNBC and anti-CRC activities, it also clarifies that our extract inhibits the proliferation of 5FU-resistant CRC cells, with comparable efficacy. However, in the case of 5FU-resistant CRC cells, RCE exerts its effect mainly through induction of autophagic cell death. It further reiterates that *Rhus coriaria* is a promising source of phytochemicals with potent anti-cancer effects, warranting further investigations.

## Data Availability

The raw data supporting the conclusions of this article will be made available by the authors, without undue reservation.
