# Supplementary material for: Rhus coriaria (Sumac) induces autophagic cell death and inhibits mTOR, p38MAPK and STAT3 pathways in 5fluorouracil-resistant colorectal cancer cells
Source: Front Pharmacol. 2025 Mar 19;16:1542204. doi: 10.3389/fphar.2025.1542204 (PMC11962434; doi:10.3389/fphar.2025.1542204)
Supplement: Supplementary file 1 [file DataSheet1.docx]

# Supplementary Material

***Rhus coriaria (Sumac) induces autophagic cell death and inhibits mTOR, p38MAPK and STAT3 pathways in 5-fluorouracil-resistant colorectal cancer cells***

Zohra Nausheen Nizami^1^, Mazoun Al Azzani^1^, Samah Khaldi^1^, Adil Farooq Wali ^2^, Rym Magramane^1^, Shamaa Abdul Samad^1^, Ali H. Eid^3^, Kholoud Arafat^4^, Yusra Al Dhaheri^1^, Samir Attoub^4^, Rabah Iratni^1*^

^1^Department of Biology, College of Science, United Arab Emirates University, Al-Ain, P.O. Box 15551, United Arab Emirates

^2^Department of Pharmaceutical Chemistry, RAK College of Pharmacy, RAK Medical and Health Sciences University, P.O. Box 11172, Ras Al Khaimah, United Arab Emirates

^3^Department of Basic Medical Sciences, College of Medicine, QU Health, Qatar University, Doha P.O. Box 2713, Qatar

^4^Department of Pharmacology & Therapeutics, College of Medicine & Health Sciences, United Arab Emirates University, Al-Ain, P.O. Box 15551, United Arab

^*^Corresponding author: R. Iratni ([R_iratni@uaeu.ac.ae](mailto:R_iratni@uaeu.ac.ae))

#

# Supplementary Figures

**
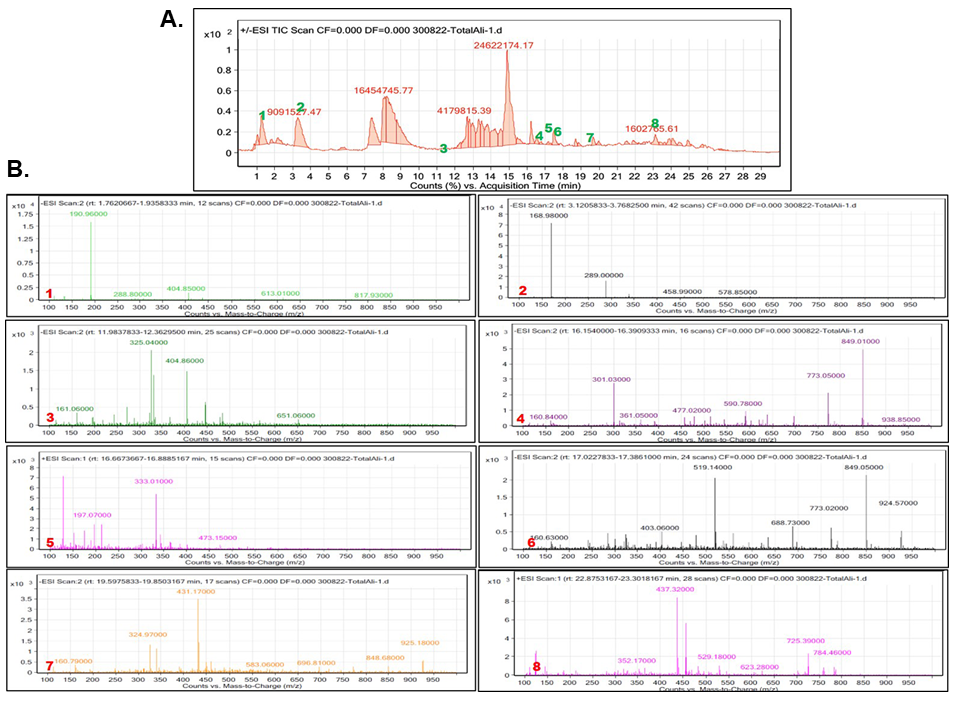
**

**Supplementary Figure 1.** The total ion current chromatograms of *Rhus coriaria* ethanolic extract (RCE) using LC-MS. (**A**) The chromatogram obtained from diode array detection at 200 and 400 nm shows eight known peaks corresponding to (**B**) (1) Quinic acid [M+H]- 190.960 m/z, (2) Gallic Acid [M+H]-168.980 m/z, (3) Coumaryl-hexoside [M+H]-325.040m/z, (4) Quercetin [M+H]- 301.010m/z, (5) Digallic Acid [M+H] +333.010 m/z, (6) Sespendole [M+H]-518.100m/z, (7) Genistin [M+H]- 431.200m/z, and (8) Phloretin 2'-glucoside [M+H] +437.300m/z.

**
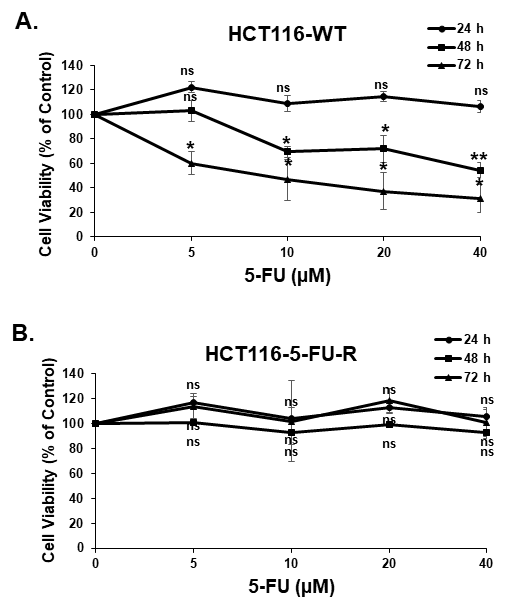
**

**Supplementary Figure 2.** 5-Fluorouracil (5FU) inhibits the viability of parental HCT116-WT cells, while having no effect on the viability of HCT116-5FU-R cells. (**A**) HCT116-WT and (**B**) HCT116-5FU-R CRC cells were treated with varying concentrations of 5‑FU for 24, 48, and 72 h to assess the effect on cell viability using MTT assay as described in the Methods section. Data is presented as the mean ± SEM; n = 3–4, in triplicate. ns: not significant, *p < 0.05, **p < 0.005, (vs. 0 µg/mL); one‑way ANOVA followed by Fisher’s LSD test.
